# Comparative Linkage Meta-Analysis Reveals Regionally-Distinct, Disparate Genetic Architectures: Application to Bipolar Disorder and Schizophrenia

**DOI:** 10.1371/journal.pone.0019073

**Published:** 2011-04-29

**Authors:** Brady Tang, Tricia Thornton-Wells, Kathleen D. Askland

**Affiliations:** 1 Biostatistics Graduate Program, Brown University, Providence, Rhode Island, United States of America; 2 Department of Molecular Physiology and Biophysics, Vanderbilt University, Nashville, Tennessee, United States of America; 3 Department of Psychiatry and Human Behavior, Butler Hospital, The Warren Alpert School of Medicine of Brown University, Providence, Rhode Island, United States of America; New England Biolabs, Inc., United States of America

## Abstract

New high-throughput, population-based methods and next-generation sequencing capabilities hold great promise in the quest for common and rare variant discovery and in the search for ”missing heritability.” However, the optimal analytic strategies for approaching such data are still actively debated, representing the latest rate-limiting step in genetic progress. Since it is likely a majority of common variants of modest effect have been identified through the application of tagSNP-based microarray platforms (i.e., GWAS), alternative approaches robust to detection of low-frequency (1–5% MAF) and rare (<1%) variants are of great importance. Of direct relevance, we have available an accumulated wealth of linkage data collected through traditional genetic methods over several decades, the full value of which has not been exhausted. To that end, we compare results from two different linkage meta-analysis methods—GSMA and MSP—applied to the same set of 13 bipolar disorder and 16 schizophrenia GWLS datasets. Interestingly, we find that the two methods implicate distinct, largely non-overlapping, genomic regions. Furthermore, based on the statistical methods themselves and our contextualization of these results within the larger genetic literatures, our findings suggest, for each disorder, distinct genetic architectures may reside within disparate genomic regions. Thus, comparative linkage meta-analysis (CLMA) may be used to optimize low-frequency and rare variant discovery in the modern genomic era.

## Introduction

The genetic architectures of many major neuropsychiatric disorders remain unresolved despite decades of linkage, fine mapping, genomewide linkage (GWLS), candidate gene association and genomewide association studies (GWAS). This lack of resolution is not due to categorical failures of any one of these methods as many independent investigations of each type have produced strong evidence of linkage or genetic association for many neuropsychiatric disorders. Rather, the apparent breakdown lies in the general lack of replication within and across methods. Importantly, although replication is the cornerstone of scientific validation, the lack of replication may be wholly consistent with the underlying genetic architectures of neuropsychiatric disorders. Each genetic method has known strengths and liabilities. Thus, rather than serving as an impediment to progress, contradictory results across studies and methods may offer valuable insights into the genetic architecture of these disorders. Our investigation focuses on bipolar disorder (BP) and schizophrenia (SCZ), which have particular public health significance due to their high heritability and prevalence, frequent treatment resistance and morbidity.

### A Note on Genetic Architecture

Thornton-Wells, et al (2004) [1] provide a critical conceptual framework for studies aiming to address genetic architecture by reviewing factors that contribute to the statistical difficulties of studying complex genetic disorders, including: allelic heterogeneity, locus heterogeneity, trait heterogeneity, phenocopy, phenotypic variability, gene-gene interactions and gene-environment interactions. They note that each of these factors complicates statistical analyses in one of two ways: 1) by creating heterogeneous, or competing, disease models or 2) by creating a multifactorial, interacting disease model. (The second model is often referred to as a ‘polygenic’ model and this term will be used hereafter.) Their definitions of allelic and locus heterogeneity and of gene-gene-interactions, in particular, are most relevant to our study.

The presence of allelic or locus heterogeneity creates heterogeneous disease models because two or more genetic variants (i.e., at two or more alleles or genes, respectively) are independently associated with the same trait in the affected population. By contrast, the presence of gene-gene interactions creates a polygenic model because two or more genetic variations interact directly or indirectly, in the individual affected persons, to alter disease risk separate from any independent effect of either variant. Thus, the former refers, implicitly, to a population-level phenomenon while the latter refers to individual-level phenomena. The authors are careful to note that each model may be relevant to different subsets of subjects affected by the same disorder and that these models are not mutually exclusive. Finally, each model will have distinct implications for the nature of the involved variants.

#### Heterogeneous Models

The degree of population-level heterogeneity and the extent of individual-level polygenicity each have implications for the expected frequencies and penetrances of the pathogenic or susceptibility variants. The population frequencies of pathogenic variants for a given disease will be *inversely* proportional to the extent of heterogeneity in the population. Under a model of robust genetic heterogeneity, then, the frequency of any single variant (e.g., allele, CNV) in the population will necessarily be low (i.e., will be a low-frequency or rare variant). Furthermore, penetrances are expected to be higher for low-frequency variants in order to give rise to a common disease in the population. (If frequencies were low and penetrances were weak, then the simultaneous expression of several rare variants would be required for disease expression in each individual and disease would necessarily be extremely rare.) The lower the frequencies of each contributing pathogenic variant in the population, the greater the number of variants necessary *in the population* to mediate risk for a common disease. Thus, a disorder dominated by a heterogeneous model is one in which many relatively rare but more highly-penetrant pathogenic variants mediate risk for the disease in the affected population.

#### Polygenic Model

The frequencies of pathogenic variants will, on the other hand, be *directly* proportional to the extent of polygenicity required for individual disease expression. Under a common disease model of robust polygenicity, the frequencies of the contributing variants in the population will need to be relatively high in order to fulfill the necessity that each affected individual carries multiple such variants. Furthermore, by virtue of their persistence in the species, common variants are expected to have low disease penetrances. Moreover, we expect that common variants of large effect would have been identified and replicated over previous decades of genetic investigation, including GWAS. Therefore, a disorder dominated by a polygenic model will be one in which several common variants of modest effect contribute to risk for disease in each affected individual.

### A Brief Comparison of Genetic Methods

#### Genetic Association

Genetic association studies, by design, select polymorphic markers within candidate genes or regions and measure the extent of allelic association with disease at those markers within a case-control or family-based design. GWAS, a much larger-scale design, agnostic with regard to candidate genes or regions, uses hundreds of thousands of tag SNPs to identify relatively small regions (tens of thousands of basepairs) likely to harbor susceptibility variants. By using common SNPs, GWAS are optimized for detection of common disease-associated alleles of modest effect.

#### Linkage Analysis

By contrast, linkage studies are family-based studies that measure the cosegregation of trait loci with genetic markers within each family. Genome-wide linkage studies (GWLS), by extension, use a large set (hundreds to thousands) of relatively evenly spaced DNA markers across the genome to detect broad regions (millions of base pairs) likely to harbor disease susceptibility loci, based on the pattern of within-family correlations between marker alleles and disease. Linkage analysis is most robust to the detection of regions harboring loci of large effect or regional clusters of uncommon/rare risk-associated loci [2], [3]. That said, the extent to which linkage analysis will produce consistent evidence of linkage across GWLS depends upon the underlying architecture of the disorder. Under a rare or private functional variant model with extensive locus heterogeneity, linkage evidence will (by definition) be modest and generally inconsistent [4], [5]).

### Relative Power

Current GWAS are optimally powered to find variants conferring relative risks of >1.1. However, as GWAS are conducted using commercial genotyping arrays designed to tag common variants, these studies are underpowered to identify low frequency risk variants even if these variants confer large (2.0) relative risks [6]. By contrast, linkage analysis is not sufficiently powered to identify alleles conferring small relative risk (i.e., 1.1–1.5) [6], [7]. However, when odds ratios at individual loci are ≥3, or there are many independent risk variants, linkage is more powerful than association [7].

### Summary of Previously Published GWAS Findings

To date, there have been 12 published GWAS [8], [9], [10], [11], [12], [13], [14], [15], [16], [17], [18], [19] and two GWAS meta-analyses [20], [21] that included subjects with bipolar disorder and 13 GWAS [9], [22], [23], [24], [25], [26], [27], [28], [29], [30], [31], [32], [33] and 3 GWAS meta-analysis [21], [29], [31] that included schizophrenic subjects. Some of these studies included mixtures of subjects with bipolar disorder, schizophrenia and/or major depressive disorder [9], [13], [20], [21].

Thus far, two independent, primary GWAS in bipolar disorder have each reported one SNP association exceeding genomewide (GW) significance (i.e., after multiple hypothesis testing corrections). First, Baum, et al [8] found GW association evidence for rs1012053, within the DGKH gene, yielding an association p-value = 1.50E-08, which exceeded *a priori* significance thresholds. Second, the Wellcome Trust Case Control Consortium (WTCCC) GWAS [14] reported association at rs420259, in the PALB2 gene, with p = 6.23E-08 exceeding the investigators' a priori threshold of p<5E-07. Though single marker results exceeding GW significance thresholds are rare, and despite minimal apparent convergence in suggestive findings across independent GWAS, four GWAS meta-analyses in bipolar (or mixed samples) have provided GW evidence for SNP association. Baum et al (2008) [34] conducted a meta-analysis of 76 SNPs with individual genotypes available from two bipolar studies [8], [14] and identified 2 SNPs (rs10791345 in JAM3, p = 1E-06; rs4806874 in SLC39A3, p = 5E-06) exceeding the study *a priori* GW significance threshold (p<7E-05). Ferreira et al (2008) [11], performed a BP meta-analysis combining their own BP sample with previously analyzed [14], [17] samples, identifying one SNP (rs10994336 in ANK3, p = 9.1E-09) that exceeded and one SNP (rs1705236 in CACNA1C, p = 7E-08) that nearly exceeded their GW threshold (p<5E-08). Wang et al (2010) [21], performed a combined BP [18] and SCZ [27], [29], [35] GWAS meta-analysis and produced 3 SNP associations (rs11789399 and rs11789407, both flanking ASTN2 gene, p = 5.56E-09 and p = 1.55E-08, respectively; and rs12201676, between GABRR1 and GABBR2 genes, p = 3.88E-08) exceeding their GW significance threshold (p<7.20E-08). Finally, Liu et al (2011) [20] performed a combined BP [14], [17] and MDD [36] GWAS meta-analysis, which identified two SNPs (rs1006737 and rs7297582, both in CACNA1C gene, p = 3.1E-08 and p = 3.4E-08, respectively) exceeding GW significance threshold (p<5E-08).

In schizophrenia, one primary GWAS [27] has reported a SNP association (rs1344706 in ZNF804A, p = 1.61E-07) exceeding the investigators' *a priori* GW significance threshold (p<5E-07). In addition to the combined BP and SCZ meta-analysis by Wang, et al [21] described above, three additional GWAS meta-analyses incorporating schizophrenic GWAS samples have produced SNP-level findings exceeding GW significance. First, Shi et al (2009) [29], performed a meta-analysis of 3 independent SCZ GWAS samples [29], [31], [37] and identified 7 GW significant SNPs spanning 209 kb on 6p22.1, and in strong LD, that contains several genes of potential biological significance. The authors could not ascertain whether the signal in this large region was driven by one or several genes, intergenic elements or by longer haplotypes that include susceptibility alleles in many genes. Second, Stefansson et al (2009) [31] performed an extended follow-up analysis to their primary GWAS (GWAS+FU) and a meta-analysis (Meta) combining results across 4 samples, finding 3 and 4 GW significant (p >1.6E-07) SNPs, respectively. Of the 7 markers attaining GW significance in either analysis, 5 (rs6913660, rs13219354, rs6932590, rs13211507, rs3131296) were located within the extended MHC region on 6p (within/near HIST1H2BJ, PRSS16, PRSS16, PGBD1, NOTCH4 genes, respectively), one (rs12807809) was 3457 bp upstream of NRGN and the other (rs9960767) was within the TCF4 gene. Finally, Athanasiu et al (2010) [22] performed a GWAS, an independent replication analysis of their top 1000 GWAS markers in another sample [38], and a combined analysis of the primary and replication samples. Their replication study produced one GW significant (a priori threshold, p<0.00024) SNP (rs7045881) association in the PLAA gene (p = 1.96E-04). Their combined analysis produced three GW significant (p<5E-05) SNP (rs7045881, rs433598, rs10761482) associations in PLAA (p = 2.2E-06), ACSM1 (p = 3.27E-06) and ANK3 (p = 7.68E-06), respectively.

In summary, then, there are several important points regarding extant bipolar and schizophrenia GWAS. First, primary GWAS in each disease have produced very few GW significant SNP findings (2 in bipolar, 1 in schizophrenia). Second, while GWAS *meta*-analyses in each disease have identified SNPs (8 in bipolar, 20 in schizophrenia) reaching GW significance thresholds in/near a handful of genes (6 genes in bipolar, 11 in schizophrenia), no two meta-analyses in a single disorder have found the same SNP (nor two SNPs within the same gene) to exceed GW significance threshold. Third, two different SNPs within the ANK3 gene (rs10994336- Ferreira et al, 2008 and rs10761482-Athanasiu et al, 2010) have produced GW significant meta-analytic evidence of association with bipolar disorder and schizophrenia, respectively. Fourth, two SNPs in the CACNA1C gene (rs1006737 and rs7297582) reached GW significance in a combined bipolar and MDD meta-analysis [20], one of which (rs1006737) surpassed GW significance, but only in the expanded reference group analysis by Ferreira et al (2008) [11].

Thus, while a small number of common variants have shown evidence for genetic association with bipolar disorder and/or schizophrenia, the vast majority of the heritability for these disorders remains unexplained by GWAS studies to date. Therefore, we suggest that extant linkage data may be an untapped and cost-efficient source of valuable information about the regional genomic architecture of low-frequency and rare variants underlying complex disorders.

### Challenges Facing Linkage Analysis

With that said, we are still left with the problem of evaluating and interpreting linkage findings in the context of the unresolved, but certainly complex, genetic architectures of neuropsychiatric disease. Generally speaking, linkage studies present two fundamental statistical barriers to replication: 1) high dimensionality relative to sample size, which may result in a significant number of false positive results and insufficient power, and 2) small effect size, likely due to a disease being caused by multiple mutations in different regions, across or within families (i.e, allelic or locus heterogeneity) [39], [40], [41]. For complex genetic diseases in particular, these problems lead to generally low linkage scores and poor agreement between different linkage studies. One approach to this problem is to use meta-analytic methods to combine the data from multiple studies. A well-constructed meta-analysis objectively integrates the results between studies, increasing power when the results are in agreement with each other [40], [41], [42], [43].

### Study Objectives: Comparative Linkage Meta-Analysis & Examination of Architectural Implications

Here we look at two meta-analytic methods: Badner and Gershon's multiple scan probability (MSP) method [41], [44] and the genome scan meta-analysis (GSMA) method [40], [45], [46]. MSP is known to have higher power to detect large effects that may have high variance (i.e., it is more dependent on effect size), while GSMA has higher power to detect effects with small variance (i.e., is more dependent on consistency of results) across independent studies [43]. A previous review of meta-analytic results derived across these methods [5] found modest consistency of results for schizophrenia and an absence of replication for bipolar, and discrepant results were attributed to differences in the datasets being analyzed by the two methods. *We were interested in identifying differences in results produced by these two methods using the same set of data as we believe that such differences may be especially useful in untangling the genetic architectures of these complex disorders.* The primary objectives of our investigation, rather than to complete comprehensive meta-analyses for these disorders, were 1) to compare the meta-analytic findings obtained under two different methods using results from the most recent published GWLS, and 2) to examine the potential implications of convergent and discrepant results for the underlying genetic architectures of bipolar disorder and schizophrenia given other genetic evidence (e.g., GWAS) available for these disorders.

Extrapolating from its methodologic strengths and weaknesses, we expect that MSP will identify relatively strong effect loci that are likely relevant to a smaller number of affected individuals. As such, we expect MSP to implicate regions likely to harbor a genetic architecture most befitting models of genetic heterogeneity. This implication should be strongest for regions in which MSP, alone, finds significant evidence of linkage.

GSMA, by contrast, is expected to identify genomic regions that most consistently harbor one or more loci related to disease across the included GWLS. Importantly, then, GSMA may implicate a region via two different mechanisms, either 1) the same locus is responsible for the region's significance or 2) separate disease-linked, low-frequency loci co-localize to the same region across GWLS samples (heretofore referred to as the single locus vs. multiple loci mechanisms, respectively). If the single locus mechanism pertains, the implicated locus will necessarily be reasonably common and, thus, be more likely to mediate smaller (i.e., modest to moderate) effects. In this case, significant GSMA findings are most likely to implicate polygenic models. If the multiple loci mechanism pertains, then the convergence of several distinct loci would have been necessary to produce regional significance, and thus, each implicated locus will be of relatively low-frequency in the sampled families and more likely of moderate to larger effect. Under the multiple loci mechanism, significant GSMA findings may be more consistent with heterogeneous models of genetic architecture. Co-localization of MSP and/or GWAS results in a significant GSMA region may then inform the relative likelihood of the two mechanisms (see Discussion).

### Hypotheses

While full testing of our more specific architectural hypotheses must await sequencing and functional investigations of genes within these regions, examining our results in light of extant genetic literature may provide additional evidence (for or against) these hypotheses. If the two meta-analytic methods are truly implicating regions with distinct architectures, we expect to find: 1) little overlap between GSMA and MSP findings for either disease; 2) relatively more agreement between the regions implicated by GWAS and those implicated by GSMA (as opposed to by MSP), particularly if the GSMA significance derives from the single locus mechanism. That said, there are caveats to the standard interpretation of GWAS that provide for an alternative expectation of agreement between GWAS and MSP findings. As will be elaborated in the Discussion, we also expect 3) more agreement between MSP and GWAS when regional clustering of rare variants creates synthetic association signals at GWAS SNP markers.

## Materials And Methods

### Data Collection

Given our comparative objectives, studies included were limited to English-language GWLS of bipolar disorder and schizophrenia catalogued in PubMed and published between 2000 and 2010. Relevant articles were obtained by searching PubMed and from the relevant references cited by authors of previous meta-analyses. (See File S1 for more detailed description of data collection, literature search screening procedures, comparison of included studies to previous GWLS meta-analyses, ethnic composition of original GWLS, marker mapping and inclusion procedures, data preprocessing, and procedures for handling missing marker data).

### Multiple-Scan Probability (MSP)

In this study we apply and contrast the results of two different methods of combining linkage studies. The first method, MSP, developed by Badner and Gershon [41], [44], modifies Fisher's method [47]. Fisher's method combines p-values from multiple tests about the same hypothesis to obtain a single test statistic:
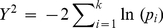
(1)


Y^2^ has a chi-square distribution (with degrees of freedom equal to twice the number of studies) under the null hypothesis and therefore yields an overall p-value that incorporates information from each individual test. When using the MSP method, this value is referred to, simply, as the MSP. Large p-values do not contribute significantly to the sum and inflate the number of degrees of freedom of the chi-square distribution, therefore increasing the MSP. As such, Fisher's method is conservative in that it takes into account evidence both for and against the null hypothesis by design. However, Fisher's method cannot be directly applied to linkage studies, because linkage evidence is often observed for broad regions and not single points [41]. This occurs as a result of association between loci which are close together on the chromosome and because studies may use different marker sets.

The modification used by MSP allows for the analysis of linkage regions by accounting (correcting) for the effects of crossover, marker spacing, family structure and original linkage methods used. After all original GWLS results are assembled, ‘corrected’ p-values (p*) are derived from each original p-value:
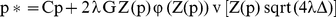
(2)p is the raw p-value, C is the number of chromosomes spanned by the region (in this case, all regions are on a single chromosome), λ is the crossover rate per Morgan, G is the region size in Morgans, Z(.) is the standard normal inverse, φ(.) is the normal density, and Δ is the marker spacing in Morgans. Next, candidate regions are identified by searching for markers with a p-value below a fixed threshold in at least one original study. Once such a marker is identified, a ‘window’ of pre-determined length is opened around that marker. The minimum observed p* falling within that window for each study is then included in the MSP calculation:
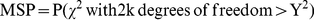
(3)by substituting p* for p_i_ in Equation 1. In this study, for the size of the linkage window and p-value threshold, we use values of 30 cM (±15 cM from triggering marker) and 0.01, respectively, following the example of Badner and Gershon (2002) [41].

Several studies utilize multiple diagnostic models. For example, a study of bipolar disorder may yield one set of linkage scores by counting only patients with bipolar I disorder as affected (narrow model), and another by counting patients with either bipolar I disorder or bipolar II disorder (broad model). This can be dealt with in MSP either by analyzing only the results based on the broadest diagnostic model used in the study (MSP-Single) or by incorporating only the most significant model's p-value and including a penalty for multiple testing (MSP-Best). We elected to complete the MSP analysis using both the MSP-Single and MSP-Best approaches in order to also evaluate and compare the results obtained with each.

It is theoretically possible for MSP to find significance because evidence for linkage was present in only one of the included studies. However, even if this is the case, the conservative design of the method allows it to provide more robust statistical evidence of linkage than that provided by the original GWLS. Discrepant linkage evidence between studies does not necessarily invalidate a finding as evidence for linkage can vary considerably depending (among other factors) on the degree of genetic heterogeneity, the proportion of parents homozygous for the susceptibility gene, ethnic stratification within the pedigree sample and ascertainment methods employed [41].

### Genome Scan Meta-Analysis (GSMA)

The GSMA method for meta-analysis of linkage studies divides each chromosome into segments of fixed length, called ’bins‘. In this study, we use 30 cM bins, following Levinson et al [40]. For each included study, bins are ranked based on the lowest p-value among markers they contain, with the bin containing the lowest p-value for that study attaining a rank of 1. Bins with tied p-values are assigned the average of their ranks [40]. The rank of each bin is summed across all studies, with studies weighted by sample size, producing a summed-rank (SR) statistic and corresponding SR p-value. Bins that consistently contain relatively low p-values will have a low SR, so that the SR p-value is a measure of the consistency of linkage evidence in that bin. Simulation studies demonstrate that SR p-values have the standard interpretation of type 1 error rate under the null hypothesis [40]. For comparability to MSP results, we conducted both GSMA-Broad and GSMA-Best analyses for each disorder. For GSMA-Broad, the lowest p-value obtained under the broadest diagnostic model employed in each original GWLS was used for determining the rank of each bin. For GSMA-Best, the best result, regardless of model, was used to determine rank.

Because GSMA is rank-based, the magnitude of a detected effect is used only to determine its rank, so that if a very large effect is observed only rarely across studies within a particular bin, GSMA will have low power to detect it. This can occur if strong linkage is only found in a region for certain populations or pertains to only one sub-phenotype. Since meta-analyses typically incorporate data from a variety of populations, it is possible to miss regions of importance to only a segment of the affected population when relying solely on GSMA. By design, GSMA will reliably fail to implicate regions in which marker rank is highly variable across [46] independent GWLS, prioritizing signal consistency over signal intensity. Additionally, GSMA relies on pre-assigned bins, and has reduced power to detect signals that fall near their boundaries, as the effect may be split between two bins. Increasing bin size may allow the effect to be captured in a single bin in specific cases, but reduces overall power, as a consistent linkage signal is unlikely to be found over a large region. For consistency, we modified the GSMA bin definitions employed by Wise et al (1999) [46] by remapping the start and end markers for each bin to deCODE.

In contrast, MSP has higher power to detect such effects, but may fail to detect small signals, even if they occur consistently. For MSP, regions of potential interest are identified based on locations of strong linkage signals from individual studies and not fixed in advance.

### Hypothesis Testing Corrections

In our results tables, we report all GSMA bins and MSP windows for which nominally-significant results were obtained on meta-analysis and indicate those results retaining significance after conservative multiple testing corrections. For the GSMA analyses, we applied standard Bonferroni corrections for 120 bins, following the example of Levinson et al (2003) [40]. For the MSP analyses, we employed the most widely-used conservative thresholds, originally proposed by Lander and Kruglyak (1995) [48] (LK-significant = 2.2E-05, LK-suggestive = 7.0E-04). Badner & Gershon (2002) [41] tested these thresholds using simulations and showed that they are conservative when using corrected probabilities (p*) and nonzero window size.

## Results

A total of 35 English-language GWLS published between 2000 and 2010 were identified through PubMed literature search. Twenty-nine were included in disease-specific MSP and GSMA analyses, including 13 for bipolar disorder [49], [50], [51], [52], [53], [54], [55], [56], [57], [58], [59], [60], [61] and 16 for schizophrenia [62], [63], [64], [65], [66], [67], [68], [69], [70], [71], [72], [73], [74], [75], [76], [77] (See Table S1 & Table S2 for full descriptions of included GWLS). The total number of studies identified, number of studies included and corresponding number of marker instances included in the meta-analyses are reported in Table 1. Multiple marker instances may occur for a single marker if the marker is used in more than one original study and/or is used to test more than one disease model in a single study.

**Table 1:** GWLS Studies and Marker Counts Included in Meta-analyses.

ss	Original GWLS Identified	Original GWLS Included	Marker Instances from Included GWLS
**Bipolar Disorder**	17	13	4640
**Schizophrenia**	18	16	12395

Results in Tables 2 and 3 display the chromosome region for the MSP window or GSMA bin, linkage window midpoint marker (MSP) or bin number (GSMA), results from MSP-Single, MSP-Best, GSMA SR (narrow model) and GSMA SR (broad model) for all windows/bins found to reach nominal significance threshold (p = 0.05) for bipolar (Table 2) and schizophrenia (Table 3) analyses. The final column in each table contains a list of the significant bipolar and schizophrenia GWAS findings, if any, for that region as reported in the Catalog of Published Genome-Wide Association Studies [78], accessed 11/17/10). Any MSP window instances with the same start/end locations necessarily produce the same results (because they are combining the same set of markers). The set of windows with non-identical start/end locations are referred to as ‘unique windows’ or, simply, ‘windows’ (as opposed to ‘window instances’).

**Table 2:** Linkage Meta-Analyses Results for Bipolar Disorder.

		p-values				
ChrBAND for MSP Window/GSMA Bin	Midpoint Marker/Bin #	MSP single	MSP best	GSMA narrow	GSMA broad	Schizophrenia (SCZ) and Bipolar Disorder (BP) GWAS Findings Overlap
2p25.3-p25.1	rs726342	**0.00564**	**0.00268**			
2p22.2-p13.3	rs195573	**0.02377**	**0.00832**			SCZ: 2p16.1 (VRK2): Stefansson - OR = 1.09, p = 3E-07
2p16.2-p11.2	rs1396798	**0.01815**	**0.00348**			**BP: 2p12 (CTNNA2): Scott - OR = 1.2, p = 3E-06; SCZ:2p16.1 (VRK2): Stefansson – OR = 1.09, p = 3E-07**
2p16.1-q11.2	D2S99	**0.07683**	**0.01821**			**BP: 2p12 (CTNNA2): Scott -OR = 1.2, p = 3E-06; SCZ:2p16.1 (VRK2): Stefansson – OR = 1.09, p = 3E-07**
3p25.3-p22.1	3.2			**0.00994**	**0.00601**	**BP: 3p22.3 (NR): Ferreira - OR = 1.18, p = 4E-06; BP: 3p24.3 (2, NR): Ferreira - OR = 1.23, p = 5E-06 & OR = 1.15, p-5E-06; BP: 3p24.3 (RFTN1): Sklar - OR = 0.79, p = 3.47E-05; SCZ&BP (meta): 3p24.2 (NR): Wang-OR = 1.28, p-1E-06)**
5p13.3-q13.3 (3 window instances)	rs336081; rs831818; rs831791	**3.63E-07** **	**3.63E-07** **			
5q14.3-q23.3	rs417670	**4.62E-08** **	**4.61E-08** **			**BP: 5q15 (MCTP1): Scott - OR = 1.21, p = 1E-07; SCZ&BP (meta): 5q21.3 (NR): Wang - OR = 1.24, p = 2E-06**); SCZ: 5q21.1 (SLCO6A1): Stefansson - 1.09, p = 1E-06
6p23-p21.1	rs2064524	**0.00050**	**0.00013**			**BP: 6p21 (NR): WTCCC - OR = 1.19, p = 4E-06**; SCZ: 6p21.32 (MHC, NOTCH): Stefansson - OR = 1.19, p = 2E-10; SCZ: 6p21.32(HLA-DQA1): Shi - OR = 1.14, p = 7E-08; SCZ: 6p22.1 (2, NR): Shi- OR = 1.28, p = 1E-08 & ISC- OR = 1.22, p = 1E-08); SCZ: 6p22.1 (MHC, PRSS16): Stefansson - OR = 1.16, p = 1E-12
7pter-p21.1	7.1			**0.05266**	**0.03724**	**SCZ&BP (meta): 7p22.3 (2, NR): Wang- OR = 1.25, p = 4E-06 & OR = 1.24, p = 2E-06; BP: 7p21.1 (SP8): Lee-OR = 1.44,p = 5E-07**
7q12.11-q31.1	7.4			**0.04438**	**0.03032**	**BP&MDD: 7q21.12 (C7orf23, DMTF1): Liu - p = 1E-06; SCZ: 7q22.1 (RELN): Shifman - OR = 1.58, p = 9E-07**
8p11.21-q21.13	rs684872	**0.02212**	**0.01304**			**SCZ&BP (meta): 8q12.1 (FAM110B):Wang - OR = 1.56, p = 2E-07**
8q12.1-q22.1	rs1364616	**0.02829**	**0.01688**			**SCZ&BP (meta): 8q12.1 (FAM110B):Wang - OR = 1.56, p = 2E-07**
8q24.13-q24.3	D8S256	0.85627	**0.03603**			**SCZ&BP (meta): 8q24.3 (NR):Wang - OR = 1.56, p = 2E-07**
MSP: 10p11.21-q22.1; GSMA (10.2): 10p14-q11.21; GSMA(10.3): 10q11.21-q22.1 (see p-values below)	rs1459990; 10.2	**0.01274**	**0.01274**	**0.01723**	**0.01266**	**BP&MDD: 10q21.2 (ANK3): Liu – p = 5E-07; BP: 10q21.2 (ANK3): Ferreira - OR = 1.45, p = 9E-09; SCZ&BP (meta): 10q11.21 (NR): Wang - OR = 1.23, p = 3E-06;** SCZ: 10q21.2 (ANK3): Athanasiu - OR = 1.16, p = 8E-06
MSP: 10q22.1-q24.1; GSMA: 10q11.21-q22.1	rs2344769; 10.3	**0.00389**	**0.00043**	**0.04708**	**0.03254**	**BP&MDD: 10q21.2 (ANK3): Liu – p = 5E-07; BP: 10q21.2 (ANK3): Ferreira - OR = 1.45, p = 9E-09; BP: 10q22.3 (NR): Ferreira - OR = 1.15, p = 8E-06; SCZ&BP (meta): 10q11.21 (NR): Wang - OR = 1.23, p = 3E-06;** SCZ: 10q21.2 (ANK3): Athanasiu - OR = 1.16, p = 8E-06
12q15-q23.2	12.4			**0.04389**	**0.03093**	**BP: 12q21.1 (TSPAN8): Sklar - OR = 0.58, p = 6.11E-07**
14q11.2-q13.1	rs1241620	**6.02E-08** **	**6.02E-08** **			**BP: 14q11.2 (NR): Ferreira - OR = 1.3, p = 5E-06; BP: 14q13.1 (NR): Ferreira - OR = 1.59, p = 5E-06; SCZ&BP (meta): 14q12 (2, NR): Wang- OR = 2.41, p-3E-06 & OR = 1.23, p = 2E-06; SCZ,BP&MDD: 14q13.1 (NPAS3): Huang - p = 4E-06**
14q12-q22.3	rs2415438	**0.00014**	**0.00014**			**BP: 14q13.1 (NR): Ferreira - OR = 1.59, p = 5E-06; SCZ,BP&MDD: 14q13.1 (NPAS3): Huang - p = 4E-06**
21q11.2-q21.3	rs12034	**0.07500**	**0.04112**			
22q12.3-q13.33	rs1473953	**0.01685**	**0.01685**			

**p<2.2E-05 (LK-significant).

*p<7.0E-04 (LK-suggestive).

**p-values in bold font indicate at least nominal significance was reached.**

**GWAS overlap in bold font indicates the overlap was with bipolar GWAS.**

**Table 3:** Linkage Meta-Analyses Results for Schizophrenia.

		p-values				
ChrBAND for MSP Window/GSMA Bin	Midpoint Marker/Bin #	MSP single	MSP best	GSMA narrow	GSMA broad	Schizophrenia (SCZ) GWAS, Bipolar Disorder (BP) GWAS and GWAS Meta-Analysis (meta) Overlap
1p13.1-q24.1#	D1S1653	0.49120	**0.00505**			
1q21.3-q24.3#	D1S1679	0.78591	**0.00233**			
1q21.3-q25.1	D1S1677	0.76803	**0.00209**			
1q23.2-q25.3	D1S196	0.75513	**0.00194**			
2p25.1-p23.2	2.2			**0.02161**	**0.01552**	**SCZ&BP (meta): 2p24.3 (NR): Wang - OR = 1.37, p = 4E-06**
2p12-q22.1	2.5			**0.03488**	**0.01497**	**SCZ&BP (meta): 2q21.2 (NAP5): Wang - OR = 1.39, p = 7E-07**; BP: 2p12 (CTNNA2): Scott - OR = 1.2, p = 3E-06 ; BP: 2q11.2 (intergenic): Scott - OR = 1.17, p = 3E-06; BP: 2q11.2 (intergenic): Ferreira - OR = 1.17, p = 3E-06
2q36.1-q37.3	D2S427	0.46951	**0.01064**			BP: 2q37.3 (NR): WTCCC - OR = 1.84, p = 7E-06
2q36.3-q37.3	D2S345	0.53986	**0.00860**			BP: 2q37.3 (NR): WTCCC - OR = 1.84, p = 7E-06
3p14.1-q12.3	3.4			**0.03722**	0.21419	
3q12.3-q22.1	3.5			**0.01405**	**0.04055**	
4p14-q13.3	4.3			**0.04692**	**0.03987**	BP: 4q12 (KIT): Scott- OR = 1.16, p = 4E-06
5p14.3-q11.2	D5S1470	0.83170	**0.02874**			
5p14.2-q11.2#	D5S426	0.83170	**0.02989**			
5q31.3-q34	D5S820	0.65163	**0.01176**			**SCZ&BP (meta): 5q33.3 (NR): Wang - OR = 1.56, p = 1E-06**
5q32-q35.1	D5S422	0.67042	**0.01599**			**SCZ&BP (meta): 5q33.3 (NR): Wang - OR = 1.56, p = 1E-06**
MSP: 6q22.31-q24.3; GSMA: 6q23.2-q25.3	D6S292; 6.5	0.92453	**0.02617**	**0.03742**	**0.02070**	BP&MDD: 6q25.2 (SYNE1): Liu - p = 1E-06; BP: 6q25.2 (NR): Ferreira - OR = 1.22, p = 4E-06
6q25.3-qter	6.6			**0.00267**	**0.01106**	
8p23.1-p21.1	D8S1145	0.44879	**0.01525**			**SCZ&BP (meta): 8p21.3 (NR): Wang - OR = 1.34, p = 4E-06**
8p23.1-p12	D8S560	0.38511	**0.01849**			**SCZ&BP (meta): 8p21.3 (NR): Wang - OR = 1.34, p = 4E-06**; BP: 8p12 (NR): Scott - OR = 1.33, p = 6E-06
8p22-p12	D8S136	0.38511	**0.01849**			**SCZ&BP (meta): 8p21.3 (NR): Wang - OR = 1.34, p = 4E-06**; BP: 8p12 (NR): Scott - OR = 1.33, p = 6E-06
8p22-p11.21#	D8S1771	0.36015	**0.01632**			**SCZ&BP (meta): 8p21.3 (NR): Wang - OR = 1.34, p = 4E-06**; BP: 8p12 (NR): Scott - OR = 1.33, p = 6E-06
8p21.3-q12.1	D8S1477	0.21596	**0.00678**			**SCZ&BP (meta): 8p21.3 (NR): Wang - OR = 1.34, p = 4E-06; SCZ&BP (meta): 8q12.1 (FAM110B): Wang - OR = 1.56, p = 2E-07**; BP: 8p12 (NR): Scott - OR = 1.33, p = 6E-06

# indicates two MSP window instances occurred.

**p-values in bold font indicate at least nominal significance was reached.**

**GWAS overlap in bold font indicates the overlap was with schizophrenia GWAS.**

### MSP Results

#### Bipolar Disorder

Of the original 4640 Bipolar GWLS marker instances subjected to our meta-analyses, 18 window instances, representing 16 unique windows, yielded at least nominally-significant findings (p<0.05) in one or both MSP meta-analysis (Table 2). Significant MSP results were found on 10 different chromosomes. A slightly greater number of windows were significant under MSP-Best (16) than MSP-Single model (13) and no windows were significant for MSP-Single (broad model) only. Additionally, results for MSP-Best were consistently equal to or more significant than results for MSP-Single, but in only one case (8q24.13-q24.3) was the difference large.

The most significant MSP results for both MSP-single and MSP-best (p = 4.61E-08) was found at 5q14.3-q23.3. Two additional windows retained significance under LK-significance criteria for both MSP-Single and MSP-Best: 14q11.2-q13.1 (p = 6.02E-08) and 5p13.3-q13.3 (p = 3.63E-07). Finally, two additional windows met LK-suggestive criteria under both models: 14q12-q22.3 (MSP-Single & MSP-Best p = 1.41E-04) and 6p23-p21.1 (MSP-Single p = 4.68E-04, MSP-Best p = 1.35E-04). Of the 16 nominally-significant MSP windows, 14 were MSP-only windows. Nine of 14 (64%) significant MSP-only windows contained 13 significant GWAS SNP and/or GWAS meta-analysis associations [11], [13], [14], [16], [21]. Of the three unique MSP windows meeting LK-significance criteria, the two most significant contained seven SNP associations from four GWAS or GWAS meta-analyses [11], [13], [16], [21] (Table 2).

#### Schizophrenia

Of the original 12395 schizophrenia GWLS marker instances subjected to meta-analyses, 22 window instances (representing 16 unique windows) yielded nominally-significant findings (p<0.05) on MSP-Best analysis. Significant MSP results were found on 5 different chromosomes. In contrast to the bipolar results, the schizophrenia MSP-Single results were dramatically weaker than those for MSP-Best and no MSP-Single result approached nominal significance (Table 3). The most significant window on MSP-Best analysis was at 1q23.2-q25.3 (p = 1.94E-03), just under LK-suggestive criteria, providing nominal evidence for linkage within the window. No schizophrenia window retained significance under LK criterion. Of the 16 nominally-significant MSP windows, 15 were MSP-only windows. Within seven of 15 (47%) MSP-only windows were three significant GWAS meta-analysis SNP associations, all of which were from the combined bipolar-schizophrenia meta-analysis by Wang, et al (2010) [21], and there was no overlap with any primary schizophrenia GWAS findings.

### GSMA Results

While several bins reached a nominal significance threshold of p<0.05 in each disorder, no bin's significance survived Bonferroni correction for 120 hypothesis tests in either disorder. That said, a pair of adjacent bins in bipolar disorder (10.2 and 10.3) and 2 pairs of adjacent bins in schizophrenia (3.4 and 3.5, 6.5 and 6.6) were implicated by nominally significant GSMA findings. As demonstrated by simulations conducted by Levinson, et al (2003) [5], [40] when adjacent clusters of bins meet GSMA significance criteria, they are unlikely to represent false positives. Furthermore, since the Bonferroni correction assumes complete independence of all tests, it is conservative in the present context.

#### Bipolar Disorder

Six of 120 bins on 4 chromosomes reached nominal significance in the bipolar GSMA analysis, under one or both models. In contrast to the bipolar MSP results, the GSMA-Broad results were consistently (though modestly) more significant than the GSMA-narrow results. The most significant bin under both broad (p = 0.0060) and narrow (p = 0.0099) analyses was bin 3.2, spanning 3p25.3-3p22.1. The second most significant bin under both broad (p = 0.0127) and narrow (p = 0.0172) models was bin 10.2, spanning 10p14-q11.21. Adjacent bin 10.3 (10q11.21-q22.1) was also nominally significant. Of the 5 nominally-significant GSMA bins, 4 (80%) were GSMA-only bins. Four of 4 (100%) significant GSMA-only bins in bipolar analysis contained 10 unique GWAS SNP associations (Table 2).

#### Schizophrenia

Seven of the 120 bins reached nominal significance in the schizophrenia analysis. In contrast to the bipolar GSMA results, some GSMA bins produced more significant findings under the GSMA-broad (3 bins) model and others under the GSMA-narrow model (4 bins). The most significant bin under both broad (p = 0.00267) and narrow (p = 0.011058) GSMA models was bin 6.6 at 6q25.3-qter. This is adjacent to another bin significant under both models: 6.5 at 6q23.2-q25.3. Additionally, adjacent bins 3.5 (3q12.3-q22.1) and 3.4 (3p14.1-q12.3) are also both significant under the narrow GSMA model. Of the 7 nominally-significant GSMA bins, 6 (85%) were GSMA-only bins. Only 2 of 6 (33%) GSMA-only bins contained 3 nominally-significant SNP associations (all from [21]). Additionally, the three bins within which these significant GWAS SNPs resided were those in which the GSMA-broad model was superior to the GSMA-narrow model.

### MSP-GSMA Overlap

#### Bipolar Disorder

Of the twenty unique genomic regions representing a significant window instance, bin or the overlap thereof (Table 2), only two regions on chromosome 10 produced partially-overlapping MSP-GSMA results. The 10p11.21-q22.1 MSP window is contained completely within the boundaries of two GSMA bins (10.2 and 10.3). Notably, this region of MSP-GSMA overlap in bipolar disorder contains the most highly-replicated gene finding in bipolar disorder to date (see Discussion). A second, nearby MSP window, 10q22.1-q24.1, also overlaps marginally with GSMA bin 10.3 at band 10q22.1.

#### Schizophrenia

Of twenty-two unique genomic regions with significant meta-analytic findings, only one region on chromosome 6 contained partially-overlapping nominally-significant MSP-GSMA results. One MSP window (6q22.31-q24.3) overlapped with a GSMA bin (6q23.2-q25.3), creating one distinct region of MSP-GSMA overlap: 6q23.2-q24.3. Interestingly, no schizophrenia GWAS has implicated this region, but a GWAS in a combined bipolar and major depressive disorder sample implicated a SNP within the non-overlapping portion of this GSMA bin (6q25.2, rs17082664-G in *SYNE1*) [20].

For both disorders, the observed absence of MSP-GSMA overlap among the significant results is further supported by the fact that most significant findings under one method did not even approach significance under the other method (See Table S3 & Table S4 for further details.).

## Discussion

Notably, 5 MSP windows retained significance after multiple testing correction in the bipolar analysis. Additionally, nominally-significant evidence for linkage was found in all primary analyses in MSP and GSMA for both disorders and several sets of adjacent bins were implicated in the GSMA analyses. The failure of GSMA (in either disease) or of MSP (in schizophrenia) to detect evidence of linkage that withstood multiple testing corrections has several potential explanations. First, there may be no true linkage for these disorders. While theoretically possible, the accumulated evidence for heritability is strong, with heritability estimates of approximately 80–85% [4], [79], making true absence of genetic linkage within families unlikely. Second, linkage (and therefore linkage meta-analysis) failed to detect robust signals because the majority of the actual contributory loci are of modest effect. In this case, linkage studies would not the method of choice for detection. We would also submit that this explanation is unlikely; while modest effect variants certainly contribute to disease susceptibility, if these were the sole contributors, we would have expected much more robust findings from studies designed especially to detect such a risk architecture (i.e., GWAS).

A third potential explanation is that the substitution of neutral p-values for missing data effectively drowned out more substantial trends in the data that would have been apparent if all marker information had been available. Thus, we would expect that the inclusion of all marker information would, by and large, lead to the preservation, and possible enhancement, of significance of the bins implicated by our GSMA analysis. This expectation bore out in our bipolar reduced (RED) post-hoc analysis wherein the identical set of 6 bins was implicated as in the full analysis, with only one change to the rank order (i.e., flipping of bins 7.4 and 12.4 between 3^rd^ and 4^th^ position). (See File S1 and Table S5 for further details on Reduced Post-Hoc Analysis.) Similarly, we expect that the inclusion of many neutral p-values in the MSP calculations is also a conservative approximation of the distribution of p-values likely to reside within a window. The RED analysis, again, demonstrated the neutralizing effect of removing studies with missing data from the analysis.

Finally, failure of most results to withstand multiple testing correction may also be the result of extensive genetic heterogeneity and contributions by rare variants in these disorders. If disease risk is largely mediated by rare or private causative variants, then individual linkage studies and conservative meta-analytic approaches may fail to produce findings exceeding standard significance thresholds, even in the presence of substantial heritability and true linkage [4]. Under such a model, only large, extended pedigrees with ‘Mendelian-like’ structures (and statistical accounting for heterogeneity) may be capable of producing robust linkage signals [80] (also see Baron, 2001 [81] for an overview of the relative merits of various sample characteristics and analytic approaches to linkage). This may, in fact, be why our strongest results were produced through MSP and by the inclusion of the Marcheco-Teruel study [57] which was conducted using a very large, extended pedigree and nonparametric methods that can account for intrafamilial heterogeneity.

### Pattern of Findings across Methods

Perhaps our most compelling finding is the unique distribution of significant regions produced by each method for each disorder (Tables 2 & 3). Given the number of MSP window-triggering marker instances in the original GWLS (SCZ = 133, BP = 56, full results not shown) included in our analyses and the full genomic coverage employed by GSMA, a substantial amount of chance overlap between methods may have been expected. However, we found minimal overlap for either disease. In fact, the vast majority of regions significant by one method did not approach significance by the other method. These findings are in keeping with the fact that the methods are robust to different types of susceptibility loci and, therefore, expected to be largely non-redundant. As we expect distinct susceptibility architectures to emerge by different genetic mechanisms and over different evolutionary timescales, we may also expect that distinct types of susceptibility loci may, largely, reside in separate genomic regions. This is consistent with our findings.

The relative dominance of MSP findings over GSMA in both disorders suggests that most linkage regions identified by our analyses likely contain low frequency or rare susceptibility loci of larger effect size while fewer contain relatively more common (i.e., low-frequency) loci of more modest effect. As most MSP regions were not implicated by GSMA, the susceptibility loci residing in these regions are likely uncommon or rare. And, to the extent that the larger effect sizes necessary for MSP detection implicate higher penetrance loci, substantial individual-level polygenicity would not be necessary for disease expression for loci in these regions. Thus, for the GWLS samples examined, the genetic architecture suggested for both disorders is one dominated by heterogeneous models, via the involvement of many low frequency or rare loci, each relevant to a subset of affected families.

A proliferation of recent genetic investigations and reviews suggest that multiple rare allelic and/or structural variants—and therefore an architecture characterized by substantial locus and allelic heterogeneity—may explain a substantial proportion of susceptibility to major neuropsychiatric disorders [3], [4], [79], [82], [83], [84], [85]. Furthermore, Cirulli & Goldstein (2010) [4] note that the diversity of linkage regions implicated across different families securely confirms high locus heterogeneity for many common diseases. In fact, their review of genetic results over the past several years suggests that common diseases may, in fact, be more similar to Mendelian diseases than is postulated by the common disease-common variant model.

### Diagnostic Models Implicated Across Meta-Analytic Methods

Interestingly, our MSP-Best analysis consistently produced stronger results than MSP-Single for both disorders. In the case of schizophrenia, in fact, all significant results were found under MSP-Best analysis only. This suggests that the regions implicated by MSP may be more likely to mediate risk for narrower diagnostic models tested across GWLS. For the GSMA analyses, the best-fitting models differed somewhat across disorders. For bipolar disorder, GSMA-Broad produced consistently stronger results than the GSMA-Best model; for schizophrenia, significance was split between GSMA-Broad (4 bins) and GSMA-Best (3 bins). Thus, across disorders, regions implicated by GSMA may be more likely to harbor loci contributing to risk for the broadest phenotypic characteristics. Again, this is consistent with our architectural hypotheses. We generally expect the most clearly defined phenotypes to be the most highly-penetrant and, therefore, more detectable by MSP. On the other hand, loci mediating broader phenotypic characteristics are expected to be less penetrant, perhaps representing common, modifier genes, best detected through GSMA.

### Comparative Linkage Meta-Analysis: Overlap with Previous Association Evidence

Short of fine mapping or, ultimately, deep sequencing of regions implicated by each method, further evidence for the architectural implications proposed here may be sought in the extant genetic literature. If differential evidence from MSP and GSMA implicates distinct genetic architectures, we may expect differences in the consistency of candidate gene association evidence within the regions implicated under each method (See File S1 for further discussion.) That said, candidate gene selection is subjective and not genomewide in scope, so such studies are certainly insufficient as a means of verifying linkage meta-analytic findings. Thus, comparison with previous GWAS findings may be most useful.

As noted in the introduction, GWAS are expected to identify regions likely to harbor common variants of modest effect. More specifically, under standard design and interpretation of GWAS methods, disease-associated GWAS SNPs are expected to be in linkage disequilibrium (LD) with, and therefore in relatively close proximity to, the putative common, functional variant. This generally accepted interpretation, however, has at least three caveats that may be relevant when comparing GWAS findings to linkage meta-analysis. First, the correlation between the strength of GWAS association signals and the extent of actual disease association at the risk locus will depend on the allele frequency of the relevant variant, degree of LD with the incorporated marker and the power of the study. Thus, modest GWAS evidence could implicate moderately-common loci and/or moderately-large effect sizes at the actual risk locus. Under these circumstances, we might expect to find regional overlap between GSMA and GWAS findings.

There are also at least two situations in which low-frequency or rare loci of relatively large effect may produce significant GWAS signals. The first would be chance oversampling of cases with the same uncommon pathogenic locus (e.g., latent family substructure). Under such circumstances, considerable inconsistencies between independent GWAS would be expected and overlap with MSP would be possible, but not predictable. The second, and perhaps more likely, situation would be cases in which signals at common SNPs were produced by ‘synthetic’ effects at multiple rare loci. As most recently discussed by Dickson, et al (2010) [86] “variants much less common than the associated one may create ‘synthetic associations’ by occurring, stochastically, more often in association with one of the alleles at the common site versus the other allele.” (p. 1) The authors systematically explore this possibility, through simulated and real GWAS data, and conclude that such synthetic associations, when present, are likely to represent effects across extremely large (i.e., 2.5 Mb) genomic intervals. Thus, if GWAS signals are attributable to such synthetic associations, we might expect overlap with MSP findings.

### Regions implicated by MSP only: GWAS overlap

Given the methodologic distinctions between MSP and GSMA, regions implicated by MSP only suggest the presence of one or more loci that are uncommon or rare in the sampled populations and of relatively large effect. Under standard assumptions of GWAS study design, we would also expect that such loci would not produce positive evidence of association in GWAS studies. In light of the above-noted caveats to standard GWAS interpretation, however, regions implicated by both MSP and GWAS (assuming both findings are true positives), in the absence of positive GSMA findings, have at least two alternative explanations. First, the regions may be implicated by both methods due to independent effects of two distinct types of loci within the region–both rare loci of large effect (+MSP) and other common, loci of modest effect (+GWAS). Second, the regions may be implicated by both methods non-independently—that is, a single locus or proximal cluster of loci that are uncommon to rare and of relatively large effect are creating both linkage and association signals (the latter via the mechanism as described above [86]).

#### Bipolar Disorder

Although overlap between GSMA with GWAS was found more consistently in bipolar disorder, there is still substantial overlap between MSP-only regions and previous GWAS findings. Additionally, other than the 5p13.3-q13.3 window, which met LK-significance criteria and did not contain a GWAS associated SNP, there appeared to be a general trend for regions with stronger MSP evidence to be more likely to overlap with previous GWAS evidence. If MSP-GWAS overlap is due primarily to independent effects at distinct locus types, then we might not expect to observe a relationship between the strength of MSP findings and the likelihood of GWAS findings. On the other hand, if the overlap is due primarily, or at least partially, to synthetic associations of rare variants, then we might predict regions with stronger MSP signals to be more likely to also contain GWAS associations, which is largely consistent with our results. The resolution of the mechanisms underlying such colocalization must await further genetic and functional analyses, but raises interesting questions about the architectures represented within regions of MSP-GWAS overlap.

#### Schizophrenia

Our finding of less MSP-GWAS overlap for schizophrenia, relative to that found in bipolar, may be somewhat surprising if only because there are more published reports of GWAS in schizophrenia than in bipolar. Three additional factors may contribute to this finding. First, smaller average population sample sizes used in schizophrenia GWAS to date will reduce power to detect association and likely contributed to the lower reported number of SNP associations for schizophrenia ([78], accessed 11/27/10). Second, the likelihood of finding GWAS overlap in schizophrenia may have been reduced by the fact that the schizophrenia GWAS samples were primarily of European ancestry, while the ethnic composition of the included schizophrenia GWLS was much more varied (See File S1 for further discussion of GWAS and GWLS characteristics).

Third, a substantial recent literature suggests that the genetic architecture of schizophrenia may be characterized by more pronounced genetic heterogeneity (via both single nucleotide and copy number variations) and contributions from rare variants [4], [26], [37], [38], [87], [88]. As noted above, GWAS is not expected to be especially robust under such an architecture. In addition, the degree to which synthetic associations of rare variants are detectable by tag SNPs used in GWAS will also depend on the MAF of the causal alleles. As demonstrated by Wang, et al (2010) [89] in their analyses of simulated and real datasets, tag SNPs significantly underestimate the true effect sizes of causal alleles and the degree of expected underestimation increases with decreasing causal MAF. Thus, regions containing only very rare causal variants are unlikely to produce GWAS signals that reach even nominal significance thresholds for reporting.

### Regions Implicated by GSMA only: GWAS overlap

Given the two alternative mechanisms by which GSMA bin significance can arise (see Introduction), the specific architectural implications of GSMA-only findings may be further refined by considering the presence (vs. absence) of co-localized GWAS findings. GSMA-only signals that overlap with positive GWAS evidence are more likely to result via the single locus mechanism, and suggest a regional architecture contributing to polygenic models. In other words, a single common locus will contribute modestly to the disease risk and, thereby, require the simultaneous effects of other loci (i.e., polygenicity) for disease expression. GSMA-GWAS overlap was found in 100% of bipolar GSMA-only bins and 33% of schizophrenia GSMA-only bins.) Moreover, in schizophrenia, GSMA-GWAS overlap was produced only by the most recent combined bipolar-schizophrenia meta-analysis [21].

GSMA-only regions without co-localized GWAS evidence may be more likely to harbor low-frequency loci of ‘moderate’ effect (i.e., variant frequencies below the GWAS detection threshold and variant penetrances below the MSP threshold.) In addition to the possible architectural causes, less frequent GSMA-GWAS overlap in schizophrenia may also result from the missing marker data for many of our included GWLS, ethnic differences between samples, or low power of the schizophrenia GWAS to date.

### Regions Implicated by both MSP & GSMA: GWAS Overlap

The regional colocalization of GSMA and MSP evidence suggests either the presence of an admixture of locus types (i.e., some low-frequency/moderate effect, others rare/strong effect) or of loci that are relatively common and relatively large in effect.

#### Bipolar Disorder

Though the two MSP windows that overlapped with significant GSMA bins were not among the bipolar windows meeting LK-significance or -suggestive criterion, their significance under both MSP models (broad and narrow), their overlap with two adjacent GSMA bins and the presence of GWAS SNP association evidence therein provides a strong complement of evidence for the localization of at least one strongly disease-linked variant. Moreover, the fact that a bipolar GWAS [15], a bipolar GWAS meta-analysis [11], a combined bipolar and MDD GWAS [20] and the recent combined GWAS meta-analysis [21] found evidence for association within the *ANK3* gene suggests that this gene is very likely among the contributors to the linkage signals. Though the convergence of MSP, GSMA and GWAS data may suggest the presence of a single, common locus of relatively large effect size, there is also evidence to support an alternate architecture at this locus.

First, at least three different SNP markers (rs9804190 [8], rs1094336 [11], and rs109433 [20]) have been implicated across bipolar (or combined) GWAS and GWAS meta-analyses, making a single common variant somewhat less unlikely. Additionally, Schulze, et al (2008) [90] performed an association analysis of two *ANK3* markers (rs9804190 and rs1094336) across three independent samples and found strong evidence supporting *ANK3* as a bipolar susceptibility locus with true, independent allelic heterogeneity; their data did not support an interacting model at these two alleles. Given our significant MSP findings in this region, and the fact that linkage is robust to allelic heterogeneity, the low GWAS odds ratio (OR) estimates for the *ANK3* gene most likely derive from the fact that genotyped tag SNPs in GWAS studies are not the actual functional variants mediating disease risk and are likely not in complete LD with the true variant(s). Additionally, the population-based design of GWAS will produce low OR estimates if disease in only a small portion of the sampled population is mediated by this locus, even if the functional variant were genotyped. Hence, the ORs produced by GWAS are unlikely to represent the true effect sizes of the functional variant(s) which are only approximated, in both location and effect, by the tagSNPs genotyped in GWAS [4], [89].

#### Schizophrenia

One significant MSP window (6q22.31-q24.3) overlapped with a significant GSMA bin (6q23.2-q25.3), creating one distinct region of MSP-GSMA overlap: 6q23.2-q24.3. Although neither the MSP window nor the GSMA bin reached LK evidence criteria, the likelihood that the GSMA results represent false positives are diminished by the fact that bin 6.6, adjacent to the overlapping bin 6.5, was also significant. Together, these findings provide a strong complement of evidence for the localization of at least one variant strongly-linked to schizophrenia. Further characterization of the true functional variant and follow-up family and population-based studies will clarify the best architectural model befitting this linked region.

### Conclusions

Our meta-analyses produced nominal evidence of linkage for bipolar disorder and schizophrenia in several genomic regions. While only windows in the bipolar MSP analysis produced evidence meeting the stringent LK-significance (3 windows) or LK-suggestive (5 windows) criteria, several other aspects of our results lend weight to our nominally-significant regions. First, we expect our results to be conservative given the likely neutralizing effects of missing data in both GSMA and MSP analyses, as suggested by comparison of full to RED bipolar analysis results in both GSMA and MSP. Second, in both bipolar disorder and schizophrenia, adjacent pairs of bins were implicated by GSMA. As noted by previous authors [45], such results are less likely to represent false positives. Third, for many of the regions implicated in our bipolar analyses (and for some implicated in the schizophrenia analyses), we found previous evidence of SNP associations from GWAS in the respective disorder. Fourth, from even a cursory review of the candidate gene literature [91], it is apparent that genes in many of our implicated regions have previous association evidence. (See File S1 for further discussion of genes within implicated regions).

Our most interesting finding is that our analysis of an identical set of marker results implicated almost entirely distinct genomic regions under the two methods. As MSP is most robust to the identification of relatively rare loci of strong effect, we expect that the regions implicated by positive findings in MSP only are most likely to harbor a genetic architecture most befitting models of genetic heterogeneity. As GSMA is most robust to the detection of genomic bins that most consistently harbor disease-related locus(i) across GWLS, we expect that the regions implicated by positive findings on GSMA only will be more likely to contain loci befitting polygenic, interacting models. Furthermore, we found a greater number of significant MSP windows than GSMA bins, suggesting that a greater number of genomic regions are likely to mediate heterogeneous architectural models while fewer are likely to mediate polygenic risk architecture.

### Future Directions

Despite strong heritability, further large scale linkage studies are unlikely to be completed given the costs of ascertaining large numbers of families and the failure of results to converge convincingly to date. Discussion abounds in the literature as to the most appropriate direction for current and upcoming genetic investigations. Some have advocated larger GWAS (i.e., with tens of thousands of cases and controls) in order to map the common variants with very small effect sizes. Advocates on the other end of the variant frequency spectrum propose the use of whole genome sequencing as the ultimate, model-free method to identify rare, highly-penetrant, functional variants. Approaches in between the extremes include: candidate gene and regulatory region sequencing, population-based approaches to linkage analysis using IBD sharing [92], using long-range haplotype phasing to select cases for sequencing near tagSNPs [86], [89], targeted re-sequencing under linkage peaks [79] and whole-exome sequencing [4]. While whole genome sequencing will soon be both feasible and affordable, the analytic burden inherent to such data may far outweigh its potential to yield meaningful discovery until further progress is made in understanding more basic aspects of risk architecture and the likely pathophysiology of these complex disorders. Interestingly, our finding may suggest that results from comparative linkage meta-analyses (CLMA) of extant data may serve to guide selection of the most appropriate type of follow-up analyses. For example, if regions mediating a heterogeneous architecture are consistently implicated across large portions of the genome, a bioinformatically-informed, whole-exome sequencing approach (e.g., one using dimensionality reduction based on genomic conservation, gene ontology or pathway involvement to prioritize likely functional variants) may be optimal for identification of rare functional variants. On the other hand, if regions likely to mediate polygenic architecture are relatively confined, denser SNP arrays and/or more targeted sequencing may be indicated.

Our analysis represents a pilot investigation to explore the use of complementary meta-analysis to illuminate genetic architecture in complex neuropsychiatric disorders. As suggested by the present results, this approach may help to prioritize regions for further analysis, depending upon the objectives of the investigators. While the identification of loci contributing modestly to population-level risk may be of greater epidemiologic relevance (i.e., contribute more to population attributable risk), the identification of disease-associated rare variants, which are much more likely to be functional, will be more helpful in elucidating underlying pathophysiological mechanisms. For these reasons, we would encourage research collaboratives and consortia with access to large numbers of full GWLS results to conduct comparative linkage meta-analyses on their own troves of linkage data.

## Supporting Information

File S1 **Supporting Information (text).**
(DOCX)

Table S1 **Included Bipolar Disorder Genome-wide Linkage Scan Characteristics.**
(DOCX)

Table S2 **Included Schizophrenia Genome-wide Linkage Scan Characteristics.**
(DOCX)

Table S3 **Average and Range of GSMA Bin Ranks for Significant MSP-Only Windows.**
(DOCX)

Table S4 **Average and Range of MSP Window Ranks for Significant GSMA-Only Bins.**
(DOCX)

Table S5 **Reduced Analysis Results for Bipolar Disorder.**
(DOCX)
