# Supplementary material for: Comparative Linkage Meta-Analysis Reveals Regionally-Distinct, Disparate Genetic Architectures: Application to Bipolar Disorder and Schizophrenia
Source: PLoS One. 2011 Apr 29;6(4):e19073. doi: 10.1371/journal.pone.0019073 (PMC3084739; doi:10.1371/journal.pone.0019073)
Supplement: File S1 — Supporting Information (text). (DOCX) [file pone.0019073.s001.docx]

**File S1. Supporting Information**

**Summary of Included GWLS**

*Data Collection*

The PubMed search was performed on September 10, 2009 using the following search terms: (schizophrenia [MeSH Terms] OR obsessive-compulsive disorder [MeSH Terms] OR bipolar disorder [MeSH Terms]) AND ("2000" [Publication Date]: "2009” [Publication Date]) AND linkage[Title]. There were too few unique GWLS of obsessive-compulsive disorder to carry out a meaningful meta-analysis, so these studies were excluded. Studies were also excluded if: 1) we were unable to obtain sufficiently complete (see below) data for all diagnostic models tested [1,2]; 2) we were unable to obtain marker identifiers to enable mapping to standard deCODE reference map [3]; or 3) there was apparent duplication in subjects included between a study and a later, included analysis [4,5,6].For some studies, we were able to obtain more complete data than presented in their respective papers through response from the authors [25,30,40,47,51,55]. See Supplementary Materials for further information on the composition of the included GWLS and overlap with previous meta-analyses.

*Screening Search Results*

Only GWLS of schizophrenia and bipolar disorder were included; studies combining schizophrenia and bipolar disorder as a single outcome were excluded. 143 records were excluded based on the article title and abstract (e.g., candidate gene association studies, non whole-genome linkage analyses). 65 were excluded based on the full text, either because the above specifications were not met, no data points with p-values above .05 were reported and a more complete dataset could not be obtained, or data could not be extracted from the article and the data could not be obtained otherwise.

*Comparison of Included Studies to Previous GWLS Meta-Analyses*

Despite the restriction to a 10 year period and the exclusions noted above, the final numbers of studies included in our bipolar (13) and schizophrenia (16) meta-analyses are comparable to most previous meta-analyses [7,8,9], with the notable exception of the Ng, et al (2008)[10] schizophrenia GSMA investigation, which included 32 GWLS. Relative to previously published meta-analyses of bipolar disorder, our meta-analyses included very few of the same studies: 2 overlap with a previous MSP analysis [11] and 3 with a previous GSMA study [9]. Relative to previously published schizophrenia GWLS meta-analyses, our study had substantial overlap (11 studies in common) with the GSMA performed by Ng, et al (2008)[10], 6 in common with another GSMA study [8] and 4 with a previous MSP analysis [11].

*Ethnic Composition of Pedigrees included in Original GWLS*

The included bipolar disorder GWLS have the following ethnic compositions: 9 European, 1 mixed European (approx 75%) and Middle Eastern (approx 25%), 3 Latin American (Costa Rica, Antioquia of Northwest Columbia, Cuba). The included schizophrenia GWLS have the following ethnic compositions: 5 European, 2 mixed European-American and African American, 3 Asian (Han Chinese, Korean, Japanese), 2 Oceanic (Indonesia, Micronesia), 2 Middle-Eastern (Arab Israeli) and 1 Latin American (Costa Rica). (See Supplementary Tables 1 and 2 for details about included GWLS.)

**Marker Mapping and Inclusion**

To minimize the likelihood of spurious meta-analytic results due to variation in the reference maps used by authors in the original studies, we elected to conduct our analyses only on the set of markers for which we were able to obtain deCODE mapping positions in the most current available deCODE map (hg19/GRCh37, STS marker and alias mapping set, downloaded from UCSC Genome on 08/21/2010). The full set of marker results obtained for the included original GWLS comprised 18156 instances. Of those, 13478 (74%) were directly mapped to deCODE by Marker ID matching. Of the remaining 4678 marker instances, 3557 (76%) could be mapped to deCODE via interpolation using available physical position information from UCSC deCODE mapping data. The remaining marker instances (1121, 6.2%) were excluded because they could not be reliably mapped. All markers from each study for which data were available were included in both sets of meta-analyses. Thus, importantly, the identical set of marker results was used for GSMA and MSP analyses.

**Data Preprocessing**

For each included marker, the corresponding p-value was generated from marker NPL and LOD scores according to their asymptotic null distributions.,., LOD scores have a distribution proportional to a chi-square distribution with one degree of freedom [12] , and NPL scores are approximately standard normal [13] . Some studies reported both NPL and LOD scores. When this occurred, we included only the NPL scores in our analysis. While LOD scores are more powerful than NPL when the true model is specified or when maximized over genetic models [14] , specification of multiple disease models creates a multiple testing problem. Studies reporting only LOD scores [15,16,17,18,19,20,21,22] were corrected for the number of combinations of disease models and diagnostic models tested.

**Missing Marker Data**

As we were primarily interested in detecting and evaluating differences between meta-analytic methods, and since we would be using the identical dataset to conduct both sets of meta-analysis, we elected to include information from as many studies as possible and to employ a uniform, conservative approach to handle missing data. Missing marker data was assigned a neutral value of 0.5. In GSMA, then, any bin for which a study did not have a p-value was assigned p= 0.5. Similarly, in MSP, each time a window is opened around a marker reaching the threshold p-value, the p-values from all other markers within that window are used to calculate the combined p-value. Thus, for each study that does not have a marker within the window, a p=0.5 value is added to the window and included in the calculation.

We included all GWLS for which we were able to, at a minimum, obtain results for all markers with a p-value < 0.05, following the example of Badner & Gershon (2002)[11]. We obtained complete marker data for 9 of 29 (31%) studies, all markers with p-values <0.5 for an additional 9 of 29 (31%) studies, and all markers with p-values <0.05 (i.e., published data) for the remaining 11 of 29 (38%) studies. The inclusion of studies for which few marker results were available is expected to result in the ‘dilution’ of the bin rankings and of the combined p-values for windows through the presence of many high (neutral) p-values. Hence, our results are expected to be extremely conservative: Any bin reaching significance likely included several significant original marker findings and any window reaching significance would have required one or more very low p-values to retain significance once combined with several neutral p-values. Moreover, as we expect the impact of missing data to be similar across methods, the inclusion of such studies should have minimal effect on our primary objective, which was to compare results obtained for each meta-analytic method. To assess the sensitivity of our analyses to the effects of a single dataset with both highly-significant results and a substantial proportion of missing data, we performed a reduced (RED) post-hoc analysis in which we excluded the Marcheco-Teruel data.

**Reduced, Post-Hoc Analysis**

In addition to the analysis of the full dataset, we performed a reduced (RED) post-hoc analysis on the bipolar GWLS in which the Marcheco-Teruel (2006) [23] dataset was removed. The RED analysis was conducted as a sensitivity analysis due to the observation that most (12 of 18) of the MSP windows reaching nominal significance on the full analysis were produced by a triggering marker from the Marcheco-Teruel [23] study. We wanted to know how results from GSMA and MSP analyses would compare if this study was removed.

As illustrated in Supplementary Table 1, all GSMA bins implicated by the full analysis were also implicated by the reduced analysis, and the magnitude of the results did not change significantly. In general, the magnitude of window significance found on RED MSP was lower than that found on the full analysis, as would be expected from the removal of highly-significant markers. However, only 5 of 12 chromosomal regions reaching the nominal significance threshold in the full MSP analysis retained nominal significance in the RED MSP analysis, and five new regions were implicated in RED that were not implicated in the full MSP analysis. Importantly, in all but one case, the chromosomal regions that lost significance with removal of the Marcheco-Teruel [23] data resulted from the removal of a Marcheco-Teruel [23]triggering marker. Each new window on 2q, 4q, 7p, 16p and 18q that reached significance only after removal of the Marcheco-Teruel [23] data was due to the window moving from nearly- to nominally-significant. All MSP windows producing nominally-significant results in the RED analysis had p-values <0.15 in the full analysis (Supp Table 1). As noted in the Methods section, the emergence of new windows with removal of Marcheco-Teruel [23] likely results from removal of neutral p-values that were assigned to regions where Marcheco-Teruel [23] results were unavailable.

**Additional Discussion of Results: MSP-GSMA Overlap**

*Bipolar Disorder*

Of the 14 MSP-only regions reaching nominal significance in bipolar disorder, the average ranking for the lowest (best)-ranked overlapping GSMA bin was 46^th^ (of 120) with a range of ranks between 9^th^ and 84^th^ (non-significant GSMA results not shown in Tables 2 & 3). Of the 4 GSMA-only bins, only one overlapped with any triggered (non-significant) MSP window (ranked 22^nd^ out of 56 triggered). The remaining 3 GSMA-only bins had no overlap with any triggered MSP window. (See Supplementary Tables 4 & 5).

*Schizophrenia*

Of the 14 MSP-only regions in schizophrenia, the average ranking for the lowest (best)-ranked overlapping GSMA bin was 55^th^ (of 120), with a range of ranks from 39^th^ to 90^th^. And for the GSMA-only bins, only 3 of 6 overlapped with any of the 133 triggered MSP windows; these had an average rank of 55^th^ (range 29-69^th^). (See Supplementary Tables 4 & 5).

**Further Support for Our Nominally-Significant Findings**

*Comparison of Results to Previous GWLS Meta-Analysis Results*

In addition to the explanations provided in the Discussion, we believe that the validity of our nominally-significant findings is further supported by the fact that several nominally-significant MSP windows and a nominally-significant bin from our analyses overlap with highly-significant findings from previous meta-analyses. In their meta-analysis, Badner & Gershon [11] identified two bipolar windows that exceeded their significance threshold, one of which overlapped with our nominally significant window at 22q12.3-q13.3. Their study also identified three significant schizophrenia windows, one of which overlapped with the 5 nominally-significant schizophrenia windows on 8p and 8pq in our study. Finally, the only bin surviving Bonferroni correction identified out of several previous GSMA analyses [8,9,10], was bin 2.5 (2p12-q22.1) in the Lewis et al (2002) [8] schizophrenia meta-analysis, which is our second most significant schizophrenia GSMA bin on the broad analysis.

*Comparison of Results to Candidate Gene Association Findings*

If differential evidence from MSP and GSMA implicates distinct genetic architectures, we will expect to find differences in the consistency of candidate gene association evidence within the regions implicated under each method. More specifically, we would expect well-replicated gene (or allelic) associations to be more likely within regions implicated by GSMA, and would expect especially strong, less replicated associations to be more likely within regions implicated by MSP. While an exhaustive search of the candidate gene literature is well beyond the scope of this paper, two genes of longstanding interest in schizophrenia may exemplify the contrast. Residing in one of our nominally-significant GSMA bins (3q12.3-q22.1) is the dopamine receptor 3 gene (DRD3). DRD3 has shown evidence (positive or trend) of association in approximately 34 of 86 (35%) studies (SzGene Database[24], accessed 11/17/10). Moreover, on meta-analysis, the odds ratios produced across different samples are quite marginal and consistent, ranging from 1.02 [25] to 1.10 [26]. On the other hand, the meta-analytic results for association studies of the rs1816072 SNP in the GABRB2 gene, which resides in two nominally-significant MSP windows (5q31.3-q34, 5q32-q35.1), showed wide variation in the odds ratios produced in case-control studies across different population samples, ranging from a low of 0.67 (CI=0.32, 1.37) in one Asian sample [27] to a high of 1.55 (CI=1.05, 2.29) in another Asian sample [28]. (See Meta-Analytic results provided in SzGene Database [24] for further examples, accessed 11/18/10.)

**Minimal Overlap between Schizophrenia Meta-Analytic Regions & GWAS**

Our finding of less MSP-GWAS overlap for schizophrenia, relative to that found in bipolar, may be somewhat surprising if only because there are more published reports of GWAS in schizophrenia (12 schizophrenia only, 2 combined with bipolar, MDD or both) than in bipolar (10 bipolar only, 3 combined). Two additional factors may contribute to the scant MSP-GWAS overlap in schizophrenia. First, the population samples used in the schizophrenia GWAS were, on average, substantially smaller than those used in bipolar GWAS. Based on case numbers reported in the Catalog of Published Genome-Wide Association Studies ([29],accessed 11/27/10), we calculated an average number of cases for published GWAS as approximately 1550 (2050, including combined studies) in bipolar vs. 1200 (1150 including combined) in schizophrenia. Smaller sample sizes will reduce power to detect disease association and are likely to contribute to the lower number of significant SNP associations reported for schizophrenia (31) relative to bipolar disorder (50) ([29], accessed 11/27/10).

**Genes implicated in GWAS and Pathways-Based Analyses**

The findings pertaining to ion channel and associated genes within the GWAS and GWAS meta-analytic studies represent a point of biological convergence and are especially noteworthy in light of findings derived by other analytic approaches to GWAS data. First, a pathways-based analysis conducted on an NIMH [30] and WTCCC [31] bipolar GWAS data demonstrated enrichment for disease-associated SNPs within the voltage-gated ion channel gene set and the broader class of ion channel genes[32]. Second, a model-free gene-centric analysis of bipolar and schizophrenia GWAS data conducted by Moskvina, et al [33], found cross-disorder gene-level replication evidence for the CACNA1C gene as well as for a related family member, CACNA1B. Finally, several genes encoding ion channel subunits and associated proteins have found suggestive evidence for association in bipolar and/or schizophrenia GWAS, including CACNG5/CACNG4 [34], KCTD12 [35], CACNB2 [35], KCNMB2 [36], and NALCN [37].

**Genes in Regions Implicated by our CLMA**

Although a full elaboration of the potentially relevant genes within the implicated regions is not possible here, a few observations may suggest further biological relevance of the implicated regions. Given the strong evidence that has accumulated supporting a role for one ankyrin protein (*ANK3*) in bipolar disorder [38,39] and schizophrenia [40], it is potentially interesting that 10 of 16 MSP windows and 3 of 6 GSMA bins implicated in the bipolar analysis also contain ankyrin and/or ankyrin repeat domain genes; in schizophrenia, there is similar overlap with 13 of 20 MSP windows and 2 of 7 GSMA bins. This overlap is particularly compelling given that the implicated *ANK3* gene (and, most likely, several other ankyrin proteins) are thought to participate in the maintenance/targeting of ion channels and cell adhesion molecules at the nodes of Ranvier and axonal initial segments, since there is also strong evidence for association of at least one voltage-gated ion channel gene (*CACNA1C*) from GWAS and meta-analyses in bipolar disorder [30,38] and schizophrenia [41,42], and suggestive evidence for other ion channel genes in GWAS [31,34,35,41,43] and pathways-based analyses[32]. The presence of ion channel genes in many of our implicated regions is also noteworthy. Twelve of 16 MSP windows and 5 of 6 GSMA bins in the bipolar analysis and 19 of 20 MSP windows and 3 of 7 GSMA bins in the schizophrenia analysis also contain one or more ion channel subunit or ion channel assembly genes. Finally, given the longstanding interest in and biological plausibility of involvement of neurotransmitter genes in mediating risk for both mood and psychotic disorders (see recent review by Serretti & Mandelli, 2008 [44]), the finding of monoaminergic and/or amino acid neurotransmitter receptor and/or transporter genes in many implicated regions is also important. In the schizophrenia analysis, 20 of 20 MSP windows and 6 of 7 GSMA bins contain neurotransmitter genes while in bipolar analyses, they were found in 11 of 16 MSP windows and 5 of 6 bins.

**REFERENCES**

1. McAuley EZ, Blair IP, Liu Z, Fullerton JM, Scimone A, et al. (2009) A genome screen of 35 bipolar affective disorder pedigrees provides significant evidence for a susceptibility locus on chromosome 15q25-26. Mol Psychiatry 14: 492-500.

2. Fullerton JM, Liu Z, Badenhop RF, Scimone A, Blair IP, et al. (2008) Genome screen of 15 Australian bipolar affective disorder pedigrees supports previously identified loci for bipolar susceptibility genes. Psychiatr Genet 18: 156-161.

3. Middleton FA, Pato MT, Gentile KL, Morley CP, Zhao X, et al. (2004) Genomewide linkage analysis of bipolar disorder by use of a high-density single-nucleotide-polymorphism (SNP) genotyping assay: a comparison with microsatellite marker assays and finding of significant linkage to chromosome 6q22. Am J Hum Genet 74: 886-897.

4. Ross J, Berrettini W, Coryell W, Gershon ES, Badner JA, et al. (2008) Genome-wide parametric linkage analyses of 644 bipolar pedigrees suggest susceptibility loci at chromosomes 16 and 20. Psychiatr Genet 18: 191-198.

5. DeLisi LE, Mesen A, Rodriguez C, Bertheau A, LaPrade B, et al. (2002) Genome-wide scan for linkage to schizophrenia in a Spanish-origin cohort from Costa Rica. Am J Med Genet 114: 497-508.

6. Faraone SV, Skol AD, Tsuang DW, Young KA, Haverstock SL, et al. (2005) Genome scan of schizophrenia families in a large Veterans Affairs Cooperative Study sample: evidence for linkage to 18p11.32 and for racial heterogeneity on chromosomes 6 and 14. Am J Med Genet B Neuropsychiatr Genet 139B: 91-100.

7. Byerley W, Badner JA (2010) Strategies to identify genes for complex disorders: a focus on bipolar disorder and chromosome 16p. Psychiatr Genet.

8. Lewis CM, Levinson DF, Wise LH, DeLisi LE, Straub RE, et al. (2003) Genome scan meta-analysis of schizophrenia and bipolar disorder, part II: Schizophrenia. Am J Hum Genet 73: 34-48.

9. Segurado R, Detera-Wadleigh SD, Levinson DF, Lewis CM, Gill M, et al. (2003) Genome scan meta-analysis of schizophrenia and bipolar disorder, part III: Bipolar disorder. Am J Hum Genet 73: 49-62.

10. Ng MY, Levinson DF, Faraone SV, Suarez BK, DeLisi LE, et al. (2009) Meta-analysis of 32 genome-wide linkage studies of schizophrenia. Mol Psychiatry 14: 774-785.

11. Badner JA, Gershon ES (2002) Meta-analysis of whole-genome linkage scans of bipolar disorder and schizophrenia. Mol Psychiatry 7: 405-411.

12. Ott J (1999) Analysis of Human Genetic Linkage; Ott J, editor. Baltimore, MD: Johns Hopkins University Press.

13. Kruglyak L, Daly MJ, Reeve-Daly MP, Lander ES (1996) Parametric and nonparametric linkage analysis: a unified multipoint approach. Am J Hum Genet 58: 1347-1363.

14. Abreu PC, Greenberg DA, Hodge SE (1999) Direct power comparisons between simple LOD scores and NPL scores for linkage analysis in complex diseases. Am J Hum Genet 65: 847-857.

15. Badenhop RF, Moses MJ, Scimone A, Mitchell PB, Ewen-White KR, et al. (2002) A genome screen of 13 bipolar affective disorder pedigrees provides evidence for susceptibility loci on chromosome 3 as well as chromosomes 9, 13 and 19. Mol Psychiatry 7: 851-859.

16. Brzustowicz LM, Hodgkinson KA, Chow EW, Honer WG, Bassett AS (2000) Location of a major susceptibility locus for familial schizophrenia on chromosome 1q21-q22. Science 288: 678-682.

17. DeLisi LE, Shaw SH, Crow TJ, Shields G, Smith AB, et al. (2002) A genome-wide scan for linkage to chromosomal regions in 382 sibling pairs with schizophrenia or schizoaffective disorder. Am J Psychiatry 159: 803-812.

18. Friddle C, Koskela R, Ranade K, Hebert J, Cargill M, et al. (2000) Full-genome scan for linkage in 50 families segregating the bipolar affective disease phenotype. Am J Hum Genet 66: 205-215.

19. Gurling HM, Kalsi G, Brynjolfson J, Sigmundsson T, Sherrington R, et al. (2001) Genomewide genetic linkage analysis confirms the presence of susceptibility loci for schizophrenia, on chromosomes 1q32.2, 5q33.2, and 8p21-22 and provides support for linkage to schizophrenia, on chromosomes 11q23.3-24 and 20q12.1-11.23. Am J Hum Genet 68: 661-673.

20. Paunio T, Ekelund J, Varilo T, Parker A, Hovatta I, et al. (2001) Genome-wide scan in a nationwide study sample of schizophrenia families in Finland reveals susceptibility loci on chromosomes 2q and 5q. Hum Mol Genet 10: 3037-3048.

21. Service S, Molina J, Deyoung J, Jawaheer D, Aldana I, et al. (2006) Results of a SNP genome screen in a large Costa Rican pedigree segregating for severe bipolar disorder. Am J Med Genet B Neuropsychiatr Genet 141B: 367-373.

22. Wijsman EM, Rosenthal EA, Hall D, Blundell ML, Sobin C, et al. (2003) Genome-wide scan in a large complex pedigree with predominantly male schizophrenics from the island of Kosrae: evidence for linkage to chromosome 2q. Mol Psychiatry 8: 695-705, 643.

23. Marcheco-Teruel B, Flint TJ, Wikman FP, Torralbas M, Gonzalez L, et al. (2006) A genome-wide linkage search for bipolar disorder susceptibility loci in a large and complex pedigree from the eastern part of Cuba. Am J Med Genet B Neuropsychiatr Genet 141B: 833-843.

24. Allen NC, Bagade S, McQueen MB, Ioannidis JP, Kavvoura FK, et al. (2008) Systematic meta-analyses and field synopsis of genetic association studies in schizophrenia: the SzGene database. Nat Genet 40: 827-834.

25. Nunokawa A, Watanabe Y, Kaneko N, Sugai T, Yazaki S, et al. (2010) The dopamine D3 receptor (DRD3) gene and risk of schizophrenia: case-control studies and an updated meta-analysis. Schizophr Res 116: 61-67.

26. Dominguez E, Loza MI, Padin F, Gesteira A, Paz E, et al. (2007) Extensive linkage disequilibrium mapping at HTR2A and DRD3 for schizophrenia susceptibility genes in the Galician population. Schizophr Res 90: 123-129.

27. Zhao C, Xu Z, Chen J, Yu Z, Tong KL, et al. (2006) Two isoforms of GABA(A) receptor beta2 subunit with different electrophysiological properties: Differential expression and genotypical correlations in schizophrenia. Mol Psychiatry 11: 1092-1105.

28. Lo WS, Harano M, Gawlik M, Yu Z, Chen J, et al. (2007) GABRB2 association with schizophrenia: commonalities and differences between ethnic groups and clinical subtypes. Biol Psychiatry 61: 653-660.

29. Hindorff LA, Junkins HA, Hall PN, Mehta JP, Manolio TA (2009) A Catalog of Published Genome-Wide Association Studies.

30. Sklar P, Smoller JW, Fan J, Ferreira MA, Perlis RH, et al. (2008) Whole-genome association study of bipolar disorder. Mol Psychiatry 13: 558-569.

31. WTCCC WTCCC (2007) Genome-wide association study of 14,000 cases of seven common diseases and 3,000 shared controls. Nature 447: 661-678.

32. Askland K, Read C, Moore J (2009) Pathways-based analyses of whole-genome association study data in bipolar disorder reveal genes mediating ion channel activity and synaptic neurotransmission. Hum Genet 125: 63-79.

33. Moskvina V, Craddock N, Holmans P, Nikolov I, Pahwa JS, et al. (2009) Gene-wide analyses of genome-wide association data sets: evidence for multiple common risk alleles for schizophrenia and bipolar disorder and for overlap in genetic risk. Mol Psychiatry 14: 252-260.

34. Curtis D, Vine AE, McQuillin A, Bass NJ, Pereira A, et al. (2010) Case-case genome-wide association analysis shows markers differentially associated with schizophrenia and bipolar disorder and implicates calcium channel genes. Psychiatr Genet.

35. Lee MT, Chen CH, Lee CS, Chen CC, Chong MY, et al. (2010) Genome-wide association study of bipolar I disorder in the Han Chinese population. Mol Psychiatry.

36. Hattori E, Toyota T, Ishitsuka Y, Iwayama Y, Yamada K, et al. (2009) Preliminary genome-wide association study of bipolar disorder in the Japanese population. Am J Med Genet B Neuropsychiatr Genet 150B: 1110-1117.

37. Wang KS, Liu XF, Aragam N (2010) A genome-wide meta-analysis identifies novel loci associated with schizophrenia and bipolar disorder. Schizophr Res 124: 192-199.

38. Ferreira MA, O'Donovan MC, Meng YA, Jones IR, Ruderfer DM, et al. (2008) Collaborative genome-wide association analysis supports a role for ANK3 and CACNA1C in bipolar disorder. Nat Genet 40: 1056-1058.

39. Schulze TG, Detera-Wadleigh SD, Akula N, Gupta A, Kassem L, et al. (2009) Two variants in Ankyrin 3 (ANK3) are independent genetic risk factors for bipolar disorder. Mol Psychiatry 14: 487-491.

40. Athanasiu L, Mattingsdal M, Kahler AK, Brown A, Gustafsson O, et al. (2010) Gene variants associated with schizophrenia in a Norwegian genome-wide study are replicated in a large European cohort. J Psychiatr Res 44: 748-753.

41. Green EK, Grozeva D, Jones I, Jones L, Kirov G, et al. (2010) The bipolar disorder risk allele at CACNA1C also confers risk of recurrent major depression and of schizophrenia. Mol Psychiatry 15: 1016-1022.

42. Nyegaard M, Demontis D, Foldager L, Hedemand A, Flint TJ, et al. (2010) CACNA1C (rs1006737) is associated with schizophrenia. Mol Psychiatry 15: 119-121.

43. Wang Y, Zhang J, Li X, Ji J, Yang F, et al. (2008) SCN8A as a novel candidate gene associated with bipolar disorder in the Han Chinese population. Prog Neuropsychopharmacol Biol Psychiatry 32: 1902-1904.

44. Serretti A, Mandelli L (2008) The genetics of bipolar disorder: genome 'hot regions,' genes, new potential candidates and future directions. Mol Psychiatry 13: 742-771.
