# Supplementary material for: Comparative Linkage Meta-Analysis Reveals Regionally-Distinct, Disparate Genetic Architectures: Application to Bipolar Disorder and Schizophrenia
Source: PLoS One. 2011 Apr 29;6(4):e19073. doi: 10.1371/journal.pone.0019073 (PMC3084739; doi:10.1371/journal.pone.0019073)
Supplement: Table S1 — Included Bipolar Disorder Genome-wide Linkage Scan Characteristics. (DOCX) [file pone.0019073.s002.docx]

**Table S1. Included Bipolar Disorder Genome-wide Linkage Scan Characteristics**

| **Reference** | **Subject Ascertainment** | **Geno-typed N** | **Number of Families** | **Number of Affecteds** | **Sample Ethnicity** | **Sample Ancestry Details** | **Diagnostic Models Tested** | **Number of Markers** | **Marker Spacing (cM)** |
| --- | --- | --- | --- | --- | --- | --- | --- | --- | --- |
| Badenhop (2002)[1] | Multigenerational pedigrees: Medium-large, multigenerational pedigrees with illness over 2+ generations & 3 affected individuals (2+ with BPI). | 231 | 13 | 69 | EUR | Australia (British, Irish descent) | I: BPI or SZ/MA; II: BIP, SZ/MA, BPI;  III: BPI, SZ/MA, BPII, UP | 400 | 10 |
| Bennett (2002)[2] |  | 509 | 151 | 288 (model I) | EUR | UK, Ireland | I*: BPI; II: BPI, BPII; III: BP1, BPII, MDDR | 398 | 9.6 |
| Cassidy (2007)[3] | ASP: BPI proband, sibling with BPI, BPII, MDDR, SZA. | 256 | 60 | 256 | EUR | Ireland | Narrow: BPI only; Broad: BPI, BPII, SA, RUP | 401 | 8.03 |
| Cheng (2006)[4] | Multiplex pedigrees & nuclear families: BPI, 1st degree relative with lifetime h/o mania (BPI or SZA-bp type). | 1060 | 154 | 601 | EUR | USA (94% White EUR, 6% other) | I: BP1 (manic syndromes); II: BP2 (I+BPII); III: BP3 (II+MDDR) | 391 | 9 |
| Etain (2006)[5] | ASPs: BPI-early AAO proband, affected sibling with BPI, BPII, SZA with no specific AAO. | 174 | 70 | 174 | EUR | EUR (GER, IRE, FRA, SCT, SWZ, ENG) | I: Broad (BPI, BPII, SZA-BP type); II: Narrow (BPI with AAO 21 yrs or less) | 384 | 9 |
| Fallin (2004)[6] | Multiply-affected pedigrees: BPI proband, 1st or 2nd degree relative with BPI, BPII. | 205 | 41 | 97 | EUR | Ashkenazi Jewish (Eastern EUR- Russia, Poland, Latvia, Lithuania, Estonia & Central EUR-Austria, Germany, Hungary, Czech Republic, Slovakia, Serbia, Romania) | I: Broad (BPI, BPII); II: Narrow (BPI) | 382 | 8.85 |
| Friddle (2000)[7] | Unilineal multiplex families: Treated BPI proband, 2+ affected (BPII with recurrent MDEs) siblings OR at least 1 affected sibling & 1 affected parent (1 parent had to be unaffected.) | 470 | 50 | 236 | EUR | USA (Maryland, Iowa) | I: BPI, BPII, SAM; II: BPI, BPII, SAM, RUP | ? | 12 |
| Herzberg (2006)[8] | Antioquia pedigrees with 3+ individuals with BP. | 91 | 6 | 50 | Latin American | Latin America (Antioquia, North West Columbia) & closely-related Central Valley of Costa Rican family (both established 16-17th centuries by admixture of males from Southern Spain and Native American (Chibchan-Paezan) females.) | BPI | 398 | 7 |
| Marcheco-Teruel (2006)[9] | Multigenerational, regionally-isolated, multiply-affected pedigree: BPI proband, family member with BPI or MDDR & positive FHx of affective disorder. | 43 | 1 | 28 | Latin American | Cuba | I: broad (BPI, MDDR); II: narrow (BPI only) | 8686 | 0.4 |
| McInnis (2003)[10] | Unilineal, multiply-affected pedigrees: BPI proband, 2+ 1st degree relative with BPI, BPII, RUP, SCZ-manic type. | 573 | 65 | 237 | EUR | USA (Maryland, Iowa) | I: narrow (BPI, BPII with RUP, SAM); II: broad: I + RUP | 842 | 5 |
| Park (2004)[11] | Multiply-affected pedigrees: BPI proband, 1+ family members with BPI or SZA-manic type. | 373 | 40 | 79 | EUR & Middle-Eastern | USA (29 EUR ancestry), Israel (11 pedigrees of Middle-Eastern extraction) | I: BP1 (BPI, SZA-mania); II: BP2 (BP1+ BPII); III: BP3 (BP2+MDDR, MDD, SZA-dep only) | 343 | 10 |
| Service (2006)[12] | Multigenerational, multiply-affected pedigree: branches with highest density of possible BPI subjects. | 82 | 1 | 25 | Latin American | Costa Rica | BPI | 2515 | ? |
| Zandi (2007)[13] | Multiply-affected pedigrees: BPI proband, 2+ 1st-degree relatives with BPI, BPII, SCZ-manic type, MDDR. | 428 | 98 | 282 | EUR | America | I: Broad (I+MDDR); II: Narrow (BPI, BPII, SAM) | 402 | 9 |

REFERENCES

1. Badenhop RF, Moses MJ, Scimone A, Mitchell PB, Ewen-White KR, et al. (2002) A genome screen of 13 bipolar affective disorder pedigrees provides evidence for susceptibility loci on chromosome 3 as well as chromosomes 9, 13 and 19. Mol Psychiatry 7: 851-859.

2. Bennett P, Segurado R, Jones I, Bort S, McCandless F, et al. (2002) The Wellcome trust UK-Irish bipolar affective disorder sibling-pair genome screen: first stage report. Mol Psychiatry 7: 189-200.

3. Cassidy F, Zhao C, Badger J, Claffey E, Dobrin S, et al. (2007) Genome-wide scan of bipolar disorder and investigation of population stratification effects on linkage: support for susceptibility loci at 4q21, 7q36, 9p21, 12q24, 14q24, and 16p13. Am J Med Genet B Neuropsychiatr Genet 144B: 791-801.

4. Cheng R, Juo SH, Loth JE, Nee J, Iossifov I, et al. (2006) Genome-wide linkage scan in a large bipolar disorder sample from the National Institute of Mental Health genetics initiative suggests putative loci for bipolar disorder, psychosis, suicide, and panic disorder. Mol Psychiatry 11: 252-260.

5. Etain B, Mathieu F, Rietschel M, Maier W, Albus M, et al. (2006) Genome-wide scan for genes involved in bipolar affective disorder in 70 European families ascertained through a bipolar type I early-onset proband: supportive evidence for linkage at 3p14. Mol Psychiatry 11: 685-694.

6. Fallin MD, Lasseter VK, Wolyniec PS, McGrath JA, Nestadt G, et al. (2004) Genomewide linkage scan for bipolar-disorder susceptibility loci among Ashkenazi Jewish families. Am J Hum Genet 75: 204-219.

7. Friddle C, Koskela R, Ranade K, Hebert J, Cargill M, et al. (2000) Full-genome scan for linkage in 50 families segregating the bipolar affective disease phenotype. Am J Hum Genet 66: 205-215.

8. Herzberg I, Jasinska A, Garcia J, Jawaheer D, Service S, et al. (2006) Convergent linkage evidence from two Latin-American population isolates supports the presence of a susceptibility locus for bipolar disorder in 5q31-34. Hum Mol Genet 15: 3146-3153.

9. Marcheco-Teruel B, Flint TJ, Wikman FP, Torralbas M, Gonzalez L, et al. (2006) A genome-wide linkage search for bipolar disorder susceptibility loci in a large and complex pedigree from the eastern part of Cuba. Am J Med Genet B Neuropsychiatr Genet 141B: 833-843.

10. McInnis MG, Lan TH, Willour VL, McMahon FJ, Simpson SG, et al. (2003) Genome-wide scan of bipolar disorder in 65 pedigrees: supportive evidence for linkage at 8q24, 18q22, 4q32, 2p12, and 13q12. Mol Psychiatry 8: 288-298.

11. Park N, Juo SH, Cheng R, Liu J, Loth JE, et al. (2004) Linkage analysis of psychosis in bipolar pedigrees suggests novel putative loci for bipolar disorder and shared susceptibility with schizophrenia. Mol Psychiatry 9: 1091-1099.

12. Service S, Molina J, Deyoung J, Jawaheer D, Aldana I, et al. (2006) Results of a SNP genome screen in a large Costa Rican pedigree segregating for severe bipolar disorder. Am J Med Genet B Neuropsychiatr Genet 141B: 367-373.

13. Zandi PP, Badner JA, Steele J, Willour VL, Miao K, et al. (2007) Genome-wide linkage scan of 98 bipolar pedigrees and analysis of clinical covariates. Mol Psychiatry 12: 630-639.
