# Supplementary material for: Comparative Linkage Meta-Analysis Reveals Regionally-Distinct, Disparate Genetic Architectures: Application to Bipolar Disorder and Schizophrenia
Source: PLoS One. 2011 Apr 29;6(4):e19073. doi: 10.1371/journal.pone.0019073 (PMC3084739; doi:10.1371/journal.pone.0019073)
Supplement: Table S2 — Included Schizophrenia Genome-wide Linkage Scan Characteristics. (DOCX) [file pone.0019073.s003.docx]

**Table S2. Included Schizophrenia Genome-wide Linkage Scan Characteristics**

| **Reference** | **Subject Ascertainment** | **Geno-typed N** | **Number of Families** | **Number of Affecteds** | **Sample Ethnicity** | **Sample Ancestry Details** | **Diagnostic Models Tested** | **Number of Markers** | **Marker Spacing (cM)** |
| --- | --- | --- | --- | --- | --- | --- | --- | --- | --- |
| Brzustowicz (2000)[1] | Described previously: Bassett et al 1993 & 1994. | 288 | 22 | 123 | EUR | Canadian-Celtic (21) or German (1) | I: Narrow (SCZ, SZA); II: Broad (I + schizophrenia-spectrum) | 381 | 9 |
| Cooper-Casey (2005)[2] | Large pedigree, 3 generations: affecteds with chronic SCZ or SZA. | 28 | 1 | 11 | Latin American | Costa Rica | I: Strict (SZA, SCZ); II: Broad (I + BP with psychosis) | 420 | 10 |
| DeLisi (2002)[3] | ASP: SZA or SZA in at least 2 siblings. | 764 | 309 | 764 | EUR & Latin American | North EUR: USA (213 N.Y.), UK (50 Oxford), Italy (33 Milan), Chile (11 Santiago), Belgium (2 Leuven) | SCZ or SZA | 396 | 10 |
| Faraone (2006)[4] | ASP: SCZ proband, 2+ SCZ siblings (Han Chinese only). | 2242 | 557 | 1207 | Asian | Han Chinese (Taiwan) | SCZ | 386 | 9 |
| Garver (2001)[5] | PBS: SCZ/SZA proband, 2+ 1st or 2nd degree relatives with SCZ spectrum. | 263 | 27 | 124 | AA & EUR | USA (23% Cauc, 77% AA) | I: Narrow (SCZ); II: Intermediate (II + SZA-dep, Cluster A PD); III: Broad (II + Psychosis NOS, MDD with psychosis) | 406 | 9 |
| Gurling (2001)[6] | Unilineal, multi(3+)-generational, multiply-affected, pedigrees with SCZ (not BP). | 182 | 13 | 68 | EUR | British (5), Icelandic (8) | I: core SCZ (SCZ, unspecified functional psychosis, SZA), 56; II: SCZ spectrum (I + schizoid, schizotypal PD), 12 | 365 | 10 |
| Hong (2009)[7] | Multiplex pedigrees: SCZ proband, 2+ 2nd degree relatives with SCZ or SZA/schizotypal PD on long-term antipsychotics. | 183 | 56 | 123 | Asian | Korea | I: broad (SCZ, SZA, schizotypal PD); II: narrow (SCZ) | 5373 | 0.69 |
| Irmansyah (2008)[8] | ASP: SCZ proband, 1+ SCZ sibling. | 540 | 124 | 267 | Oceania | Indonesia | SCZ only | 402 | na |
| JSSLG (2003)[9] | ASP: SCZ proband, 1+ SCZ sibling. | 338 | 130 | 296 | Asian | Japan | SCZ only | 417 | 10 |
| Lerer (2003)[10] | Multiply-affected Arab-Israeli pedigrees: SCZ proband, 1+ 1st degree relative with SCZ or SZA. | 155 | 21 | 75 | Middle-Eastern | Arab Israeli | I: narrow (SCZ, SZA-dep); II: core (I + SZA-manic or dep & manic + unspecified fx psychosis); III: broad (II + nonaffective psychosis, schizotypal, affective psychosis, MDD psychotic) | 350 | 10.3 |
| Lindholm (2001)[11] | Multigenerational, multiply-affected pedigree: family members with SCZ, SZA, Psychosis NOS. | 43 | 1 | 43 | EUR | Northern Sweden | I: SCZ (SCZ); II: SAD (I + SZA-depressed type); III: Brd1 (II + Psychosis NOS); IV: Brd2 (III + SZA-bipolar type) | 371 | 10 |
| Paunio (2001)[12] | Multiply-affected pedigrees with SCZ (Finnish). | 1265 | 238 | 591 | EUR | Finnish | I: LC1 (SCZ); II: LC2 (I + SZA); III: LC3 (II + SCZ spectrum, including PDs); IV: LC4 (all individuals with severe major affective disorders) | 315 | 10-20 |
| Straub (2002)[13] | Multiplex pedigrees: SCZ proband, relatives with SCZ spectrum/other disorders. | 1425 | 270 | 1172 | EUR | Irish | I: Narrow (core SCZ, poor-outcome SZA); II: Intermediate (I+ schizotypal PD, other nonaffective psychotic disorders); III: Broad (II + psychotic affective illness, paranoid, avoidant & schizoid PD); IV: Very Broad (III+nonpsychotic affective, anxiety, EtOHism, other PDs) | 684 | 5.3 |
| Suarez (2006)[14] | ASP: SCZ proband, 1+ sibling with SCZ or SZA. | 1380 | 409 | 1006 | EUR & AA | EUR ancestry (279), AA (124) | SCZ | 400 | 9 |
| Teltsh (2008)[15] | Multiplex Israeli-Arab pedigree with SCZ spectrum disorders. | 56 | 1 | 24 | Middle-Eastern | Arab Israeli | SCZ spectrum (SCZ-14, SZA-dep type-3, SZA-manic type-1, psychosis unspecified-3, schizotypal PD-2, MDDpsychotic-1) | 346 | 10.3 |
| Wijsman (2003)[16] | Multigenerational, regionally-isolated, multiply-affected pedigree with SCZ or SZA. | 212 | 2 | 30 | Oceania | Micronesian Islandic population of Kosrae | SCZ (28), SZA (2) | 398 | 8.9 |

**REFERENCES**

1. Brzustowicz LM, Hodgkinson KA, Chow EW, Honer WG, Bassett AS (2000) Location of a major susceptibility locus for familial schizophrenia on chromosome 1q21-q22. Science 288: 678-682.

2. Cooper-Casey K, Mesen-Fainardi A, Galke-Rollins B, Llach M, Laprade B, et al. (2005) Suggestive linkage of schizophrenia to 5p13 in Costa Rica. Mol Psychiatry 10: 651-656.

3. DeLisi LE, Mesen A, Rodriguez C, Bertheau A, LaPrade B, et al. (2002) Genome-wide scan for linkage to schizophrenia in a Spanish-origin cohort from Costa Rica. Am J Med Genet 114: 497-508.

4. Faraone SV, Hwu HG, Liu CM, Chen WJ, Tsuang MM, et al. (2006) Genome scan of Han Chinese schizophrenia families from Taiwan: confirmation of linkage to 10q22.3. Am J Psychiatry 163: 1760-1766.

5. Garver DL, Holcomb J, Mapua FM, Wilson R, Barnes B (2001) Schizophrenia spectrum disorders: an autosomal-wide scan in multiplex pedigrees. Schizophr Res 52: 145-160.

6. Gurling HM, Kalsi G, Brynjolfson J, Sigmundsson T, Sherrington R, et al. (2001) Genomewide genetic linkage analysis confirms the presence of susceptibility loci for schizophrenia, on chromosomes 1q32.2, 5q33.2, and 8p21-22 and provides support for linkage to schizophrenia, on chromosomes 11q23.3-24 and 20q12.1-11.23. Am J Hum Genet 68: 661-673.

7. Hong KS, Won HH, Cho EY, Jeun HO, Cho SS, et al. (2009) Genome-widely significant evidence of linkage of schizophrenia to chromosomes 2p24.3 and 6q27 in an SNP-Based analysis of Korean families. Am J Med Genet B Neuropsychiatr Genet 150B: 647-652.

8. Irmansyah SG, Schwab, Heriani HY, Handoko A, Kusumawardhani I, et al. (2008) Genome-wide scan in 124 Indonesian sib-pair families with schizophrenia reveals genome-wide significant linkage to a locus on chromosome 3p26-21. Am J Med Genet Part B (Neuropsychiatric Genetics) 147B: 1245-1252.

9. JSSLG (2003) Initial genome-wide scan for linkage with schizophrenia in the Japanese Schizophrenia Sib-Pair Linkage Group (JSSLG) families. Am J Med Genet B Neuropsychiatr Genet 120B: 22-28.

10. Lerer B, Segman RH, Hamdan A, Kanyas K, Karni O, et al. (2003) Genome scan of Arab Israeli families maps a schizophrenia susceptibility gene to chromosome 6q23 and supports a locus at chromosome 10q24. Mol Psychiatry 8: 488-498.

11. Lindholm E, Ekholm B, Shaw S, Jalonen P, Johansson G, et al. (2001) A schizophrenia-susceptibility locus at 6q25, in one of the world's largest reported pedigrees. Am J Hum Genet 69: 96-105.

12. Paunio T, Ekelund J, Varilo T, Parker A, Hovatta I, et al. (2001) Genome-wide scan in a nationwide study sample of schizophrenia families in Finland reveals susceptibility loci on chromosomes 2q and 5q. Hum Mol Genet 10: 3037-3048.

13. Straub RE, MacLean CJ, Ma Y, Webb BT, Myakishev MV, et al. (2002) Genome-wide scans of three independent sets of 90 Irish multiplex schizophrenia families and follow-up of selected regions in all families provides evidence for multiple susceptibility genes. Mol Psychiatry 7: 542-559.

14. Suarez BK, Duan J, Sanders AR, Hinrichs AL, Jin CH, et al. (2006) Genomewide linkage scan of 409 European-ancestry and African American families with schizophrenia: suggestive evidence of linkage at 8p23.3-p21.2 and 11p13.1-q14.1 in the combined sample. Am J Hum Genet 78: 315-333.

15. Teltsh O, Kanyas K, Karni O, Levi A, Korner M, et al. (2008) Genome-wide linkage scan, fine mapping, and haplotype analysis in a large, inbred, Arab Israeli pedigree suggest a schizophrenia susceptibility locus on chromosome 20p13. Am J Med Genet B Neuropsychiatr Genet 147B: 209-215.

16. Wijsman EM, Rosenthal EA, Hall D, Blundell ML, Sobin C, et al. (2003) Genome-wide scan in a large complex pedigree with predominantly male schizophrenics from the island of Kosrae: evidence for linkage to chromosome 2q. Mol Psychiatry 8: 695-705, 643.
