# Supplementary material for: Comparative Linkage Meta-Analysis Reveals Regionally-Distinct, Disparate Genetic Architectures: Application to Bipolar Disorder and Schizophrenia
Source: PLoS One. 2011 Apr 29;6(4):e19073. doi: 10.1371/journal.pone.0019073 (PMC3084739; doi:10.1371/journal.pone.0019073)
Supplement: Table S3 — Average and Range of GSMA Bin Ranks for Significant MSP-Only Windows. (DOCX) [file pone.0019073.s004.docx]

**Table S3. Average and Range of GSMA Bin Ranks for Significant MSP-Only Windows**

| MSP Analysis | Number of Bins for Comparison | Average Rank of Best Overlapping Bin | Range of Ranks for Overlapping Bins |
| --- | --- | --- | --- |
| Bipolar | 120 | 46th | 9th to 84th |
| Schizophrenia | 120 | 55th | 39th to 90th |
