# Supplementary material for: Comparative Linkage Meta-Analysis Reveals Regionally-Distinct, Disparate Genetic Architectures: Application to Bipolar Disorder and Schizophrenia
Source: PLoS One. 2011 Apr 29;6(4):e19073. doi: 10.1371/journal.pone.0019073 (PMC3084739; doi:10.1371/journal.pone.0019073)
Supplement: Table S4 — Average and Range of MSP Window Ranks for Significant GSMA-Only Bins. (DOCX) [file pone.0019073.s005.docx]

**Table S4. Average and Range of MSP Window Ranks for Significant GSMA-Only Bins**

| GSMA Analysis | Number of Triggered Windows for Comparison | Number of Triggered Windows Overlapping any Significant Bin | Average Rank of Best Overlapping Window | Range of Ranks for Overlapping Windows |
| --- | --- | --- | --- | --- |
| Bipolar | 56 | 1 of 4 | 22nd | NA |
| Schizophrenia | 133 | 3 of 6 | 55th | 29th to 69th |
