# Supplementary material for: Comparative Linkage Meta-Analysis Reveals Regionally-Distinct, Disparate Genetic Architectures: Application to Bipolar Disorder and Schizophrenia
Source: PLoS One. 2011 Apr 29;6(4):e19073. doi: 10.1371/journal.pone.0019073 (PMC3084739; doi:10.1371/journal.pone.0019073)
Supplement: Table S5 — Reduced Analysis Results for Bipolar Disorder. (DOCX) [file pone.0019073.s006.docx]

**Table S5. Reduced Analysis Results for Bipolar Disorder**

| **Reduced Analysis** | | | | | | | **Full Analysis (for Comparison)** | | | | |
| --- | --- | --- | --- | --- | --- | --- | --- | --- | --- | --- | --- |
|  |  | **p-values** | | | |  | **p-values** | | | |  |
| **MSP Midpoint Marker/ GSMA bin** | **Window/Bin ChrBAND** | **GSMA narrow** | **GSMA broad** | **MSP single** | **MSP best** | **Study that generated Window** | **GSMA narrow** | **GSMA broad** | **MSP single** | **MSP best** | **Study that generated Window** |
| D2S99 | 2p16.1-p11.2 |  |  | 0.54828 | **0.03495** | McInnis[1] |  |  | 0.07683 | **0.01821** | McInnis[1] |
| D2S1776 | 2q23.3-q32.1 |  |  | 0.38533 | **0.03866** | Zandi[2] |  |  | 0.49754 | 0.09804 | NA |
| 3.2 | 3p25.3-p22.1 | **0.00975** | **0.00590** |  |  |  | **0.00994** | **0.00601** |  |  | NA |
| D4S1629 | 4q31.1-q34.1 |  |  | 0.44163 | **0.01960** | McInnis[1] |  |  | 0.55585 | 0.15424 | NA |
| D4S3051 | 4q35.1-q35.2 |  |  | 0.74159 | **0.04576** | McInnis[1] |  |  | 0.82816 | 0.15197 | NA |
| rs831818 | 5p13.2-q13.3 |  |  | 0.16201 | **0.04849** | Service[3] |  |  | **3.63E-07** | **3.63E-07** | Service[3] |
| D7S513 7.1 | 7p22.2-p15.3 7pter-p21.1 | 0.05276 | **0.03719** | 0.61822 | **0.01960** | Friddle[4] | 0.05266 | **0.03724** | 0.72388 | 0.06684 | NA  NA |
| 7.4 | 7q12.11-q31.1 | **0.04435** | **0.03024** |  |  |  | **0.04438** | **0.03032** |  |  | NA |
| D8S256 | 8q24.13-q24.3 |  |  | 0.77738 | **0.03975** | Friddle[4] |  |  | 0.85628 | 0.03603 | Friddle[4] |
| 10.2 | 10p14-q11.21 | **0.01713** | **0.01263** |  |  |  | **0.01723** | **0.01266** |  |  | NA |
| 10.3 | 10q11.21-q22.1 | **0.04761** | **0.03284** |  |  |  | **0.04708** | **0.03254** |  |  | NA |
| 12.4 | 12q15-q23.2 | **0.0439** | **0.03084** |  |  |  | **0.04389** | **0.03093** |  |  | NA |
| D16S3103 | 16p13.2-p12.1 |  |  | 0.26990 | **0.02266** | Cheng[5] |  |  | 0.36992 | 0.09486 | NA |
| D18S878 | 18q21.2-q22.3 |  |  | 0.19110 | **0.01960** | McInnis[1] |  |  | 0.27542 | 0.06100 | NA |
| rs12034 | 21q11.2-q21.3 |  |  | **0.04390** | 0.10041 | Service[3] |  |  | 0.07497 | **0.04112** | Service[3] |

**REFERENCES**

1. McInnis MG, Lan TH, Willour VL, McMahon FJ, Simpson SG, et al. (2003) Genome-wide scan of bipolar disorder in 65 pedigrees: supportive evidence for linkage at 8q24, 18q22, 4q32, 2p12, and 13q12. Mol Psychiatry 8: 288-298.

2. Zandi PP, Badner JA, Steele J, Willour VL, Miao K, et al. (2007) Genome-wide linkage scan of 98 bipolar pedigrees and analysis of clinical covariates. Mol Psychiatry 12: 630-639.

3. Service S, Molina J, Deyoung J, Jawaheer D, Aldana I, et al. (2006) Results of a SNP genome screen in a large Costa Rican pedigree segregating for severe bipolar disorder. Am J Med Genet B Neuropsychiatr Genet 141B: 367-373.

4. Friddle C, Koskela R, Ranade K, Hebert J, Cargill M, et al. (2000) Full-genome scan for linkage in 50 families segregating the bipolar affective disease phenotype. Am J Hum Genet 66: 205-215.

5. Cheng R, Juo SH, Loth JE, Nee J, Iossifov I, et al. (2006) Genome-wide linkage scan in a large bipolar disorder sample from the National Institute of Mental Health genetics initiative suggests putative loci for bipolar disorder, psychosis, suicide, and panic disorder. Mol Psychiatry 11: 252-260.
